# The IQA Energy Partition in a Drug Design Setting: A Hepatitis C Virus RNA-Dependent RNA Polymerase (NS5B) Case Study

**DOI:** 10.3390/ph15101237

**Published:** 2022-10-08

**Authors:** César A. Zapata-Acevedo, Paul L. A. Popelier

**Affiliations:** 1 Department of Chemistry, University of Manchester, Oxford Road, Manchester M13 9PL, UK; 2 Instituto de Química, Universidad Nacional Autónoma de México, Circuito Exterior s/n, Ciudad Universitaria, Coyoacán, Ciudad de México 04510, Mexico; 3 Tecnológico de Monterrey, Campus Santa Fe, Av. Carlos Lazo 100, Santa Fe, La Loma, Álvaro Obregón, Ciudad de México 01389, Mexico

**Keywords:** interacting quantum atoms, quantum chemical topology, quantum theory of atoms in molecules, fragment-based drug design, NS5B, hepatitis C virus, relative energy gradient method

## Abstract

The interaction of the thumb site II of the NS5B protein of hepatitis C virus and a pair of drug candidates was studied using a topological energy decomposition method called interacting quantum atoms (IQA). The atomic energies were then processed by the relative energy gradient (REG) method, which extracts chemical insight by computation based on minimal assumptions. REG reveals the most important IQA energy contributions, by atom and energy type (electrostatics, sterics, and exchange–correlation), that are responsible for the behaviour of the whole system, systematically from a short-range ligand–pocket interaction until a distance of approximately 22 Å. The degree of covalency in various key interatomic interactions can be quantified. No exchange–correlation contribution is responsible for the changes in the energy profile of both pocket–ligand systems investigated in the ligand–pocket distances equal to or greater than that of the global minimum. Regarding the hydrogen bonds in the system, a “neighbour effect” was observed thanks to the REG method, which states that a carbon atom would rather not have its covalent neighbour oxygen form a hydrogen bond. The combination of IQA and REG enables the automatic identification of the pharmacophore in the ligands. The coarser Interacting Quantum Fragments (IQF) enables the determination of which amino acids of the pocket contribute most to the binding and the type of energy of said binding. This work is an example of the contribution topological energy decomposition methods can make to fragment-based drug design.

## 1. Introduction

The energy partitioning of quantum systems at the atomic level has been used for decades to provide chemical insight into a variety of chemical phenomena, such as rotation barriers, hydrogen bonding, halogen bonding, or reactions. A modern addition to the basket of energy decomposition schemes is topological energy partitioning [1,2], which does not suffer from the typical problems. The best known topological energy partitioning method is interacting quantum atoms (IQA) [3], which is used here to analyse the energy landscape of systems consisting of the hepatitis C virus NS5B protein’s thumb allosteric site II and two drug candidates discovered by Antonysamy and coworkers [4].

IQA is an internal energy partition method based on quantum chemical topology (QCT) [5], which is a generalisation of the quantum theory of atoms in molecules (QTAIM) [6]. QCT is an interpretative paradigm that employs the concept of a (gradient) vector field of 3D quantum mechanical functions, such as the electron density, to partition a system. In the case of electron density, QCT reverts to QTAIM. Quantum topological atoms are three-dimensional fragments of electron density, with a well-defined kinetic energy, and with a finite volume. These atoms do not overlap and do not leave gaps between them either.

Since its introduction in 2005, IQA has successfully explained [7] quantum and electrostatic effects without the need for a reference system [3]. This energy partition rigorously defines primary types of energy from all chemical phenomena as follows: intra-atomic (or steric [8], including kinetic energy), electrostatic, exchange, and correlation. IQA has allowed for valuable insight to be discovered in a wide variety of chemical systems [9], such as lone-pair–π interactions [10], the transferability of properties between equivalent atoms in subsequently longer oligopeptides [11], resonance-assisted hydrogen bonds [12], the exponential nature of the interatomic repulsion energy, the special type of halogen bonding in which fluorine is involved [13], how water clusters have a bifunctional catalyst role in the formation of acid rain [14], the preference of tetracoordinated hydrogen bonds in large water clusters [15], and the interactions between two perpendicular hydrogen molecules [16], to mention a few.

Fragment-based drug design takes a small drug candidate as a scaffold and adds functional groups to increase its affinity for the protein pocket that it should bind to [17]. Since this kind of rational drug design [18,19,20,21] involves different stages, it allows for IQA to analyse the differences between them. QCT applications in drug design have been underexplored because of the required size of a chemical system to obtain meaningful insight, but advances in software and the escalating increase in computing power have made it feasible to start applying it to biologically relevant scales [22].

We have developed a method called relative energy gradient (REG) [23], which with minimal, if any, assumptions computes chemical insight into a given phenomenon, such as a rotation barrier. Although it is a general method, REG typically operates on IQA energy contributions. It has been applied to case studies such as the fluorine gauche effect, the planar and perpendicular torsional energy barriers in biphenyls, halogen bond directionality, the reaction mechanism in HIV-1 protease, and model S_N_2 reactions, to name a few. Although REG will be explained in the Section 3, we already highlight that it needs a sequence of molecular configurations, governed by some control coordinate. In essence, REG then looks at the change in energy occurring in this sequence of configurations. REG compares the energy change in the total system (i.e., the enzyme pocket and ligand in this work) with that of a given atom or pair of interacting atoms. By comparing the total energy gradient with the atomistic energy gradient, REG ranks all atomic energy contributions according to their importance in explaining the nature of the total energy change. This is how chemical insight into the phenomenon at hand is obtained. In the current context of the binding of a drug candidate to an enzyme, the ranking of REG values will highlight the pharmacophore of the drug candidate and the atoms of the protein that it strongly binds to.

## 2. Systems

### 2.1. Overall Context

Hepatitis C is a blood-borne disease caused by a positive-strand RNA virus known as hepatitis C virus (HCV). It is responsible for acute and chronic hepatitis infection, and a significant number of the infected will develop liver cirrhosis or liver cancer. Given that nearly 175 million people worldwide, around 3% of the human population, are infected, it is considered a public health problem [24]. The non-structural 5 B (NS5B) RNA-dependent RNA polymerase is one of the major targets of antiviral drugs currently used in hospitals against HCV [25]. Additionally, known as the HCV NS5B protein, it plays a central role in virus replication because it catalyses the synthesis of a negative RNA strand from the original genome. This strand later becomes a template for the synthesis of more positive-strand RNA, allowing the infection to spread.

As the NS5B protein does not have a counterpart in mammalian cells, it is an attractive drug target. Several small molecules acting as allosteric inhibitors for this protein have been published to date [26,27,28], reflecting the continuing priority the NS5B has as a drug target for both pharmaceutical industries and academia. The allosteric sites in the NS5B thumb domain have attracted attention as drug targets, as their non-nucleoside inhibitors are particularly promising [29]. Crystal structures of the HCV NS5B protein complexed with non-nucleoside inhibitors have been uploaded to the Protein Data Bank, thanks to which the allosteric inhibition has been studied with computer-aided drug design approaches [30].

The thumb site II, also known as Thumb Pocket 2 (denoted TP-2) is a narrow hydrophobic cavity near the base of the thumb domain [25], approximately 30 to 35 Å away from the active site [31]. In their fragment-based drug design study, Antonysamy et al. [4] discovered novel non-nucleoside inhibitors for the thumb site II of the NS5B protein. These inhibitors shared a number of common features because of the fragment-based drug design procedure; a compound with successful binding became the scaffold for a new batch of candidates where extra functional groups were added. The process keeps the initial interactions and improves the affinity by increasing the number of interactions between the protein pocket and new parts of the inhibitor. Several different functional groups are linked to achieve high-affinity binding [32].

The structures used in this work are neither the initial nor the final compounds of the affinity optimisation procedure, but their crystal structures were uploaded to the Protein Data Bank (PDB), and most importantly, the ligands share the binding pose. Table 1 provides a summary of the structure and properties of the ligands. While measuring the equilibrium binding affinities for the initial drug candidates in their study, the authors found the frequent presence of aryl or heteroaryl bromide, and a preponderance of carboxylic acids [4]. The importance of carboxylic acids for NS5B non-nucleoside inhibitors was also found in previous reports [33]. After several initial fragments in the screening library, the drug designers settled on the 5-bromoanthranilic acid as a scaffold for the drug candidates, and the addition of a 3,5-*cis*-dimethylpiperidine making critical interactions with the protein, creating the 3CJ2 drug candidate. The compound was improved by attaching a succinic acid group to the aniline. The new compound, ligand 3CJ4, showed a 17-fold improvement over the parent, while maintaining the binding pose.

**Figure 1:**
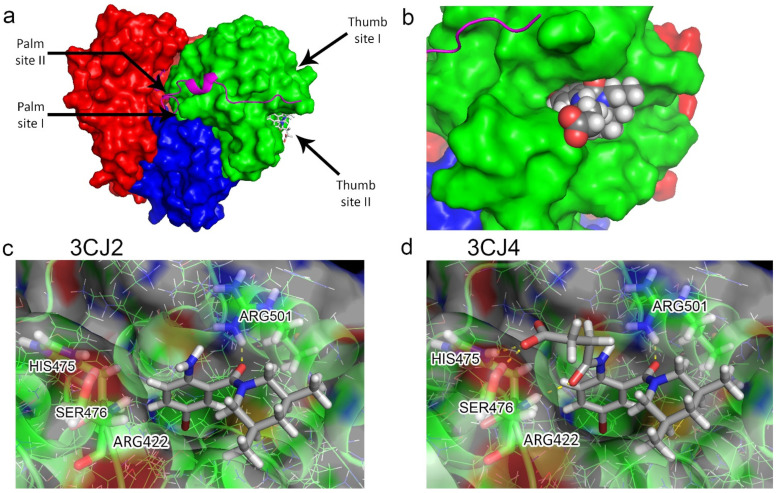
(**a**) Surface diagram showing the domains of the HCV NS5B protein [35,36]. (**b**) Location of the allosteric site II in the thumb domain of the HCV NS5B protein. (**c**) Binding pose of the drug candidate 3CJ2, with the bromine atom (brown) from the bromo-aryl moiety pointing towards the bottom of a non-polar pocket, and the amide oxygen interacting with Arg501. (**d**) Binding pose of the improved 3CJ4 drug candidate, in which all interactions from the parent are maintained. Succinic acid is added, which in turn undergoes polar interactions with other residues in the site. Pictures were generated using PyMOL [37].

Figure 1 shows the TP-2 in the NS5B protein and the two ligands. The initial 3CJ2 ligand binds to the allosteric site TP-2 via one hydrogen bond with Arg501 (a common interaction for TP-2 site inhibitors [34]). There is electrostatic attraction between the Arg422 at the bottom of the allosteric site and the partial negative charge on the bromine in the bromo-aryl moiety, and non-polar interactions between the rest of the pocket and the aromatic ring. The 3CJ4 ligand keeps all the previous interactions, plus three new hydrogen bonds: to Ser476 (one to the backbone and one to the sidechain) and to the sidechain of His475.

### 2.2. System Details

**Figure 2:**
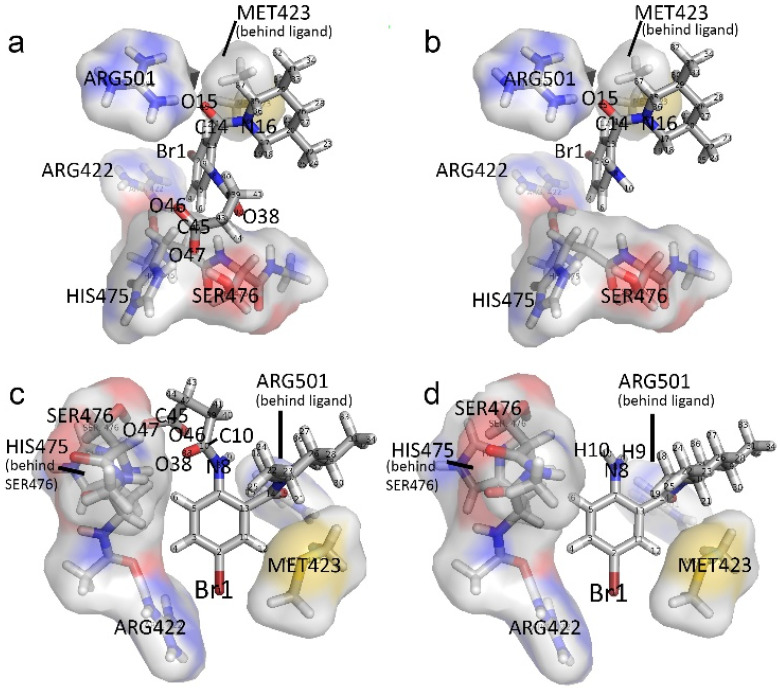
The 117- and 107-atom systems used for the REG-IQA analysis. The system involves five residues, three of which are charged. The residues were selected because (i) they make critical interactions with the ligand, (ii) affect the charge, and (iii) are the most important to keep the shape of the site in the model. (**a**,**b**) Top view of the allosteric site representation for (**a**) 3CJ4 and (**b**) 3CJ2, respectively. (**c**,**d**) Side view of the same systems. The Arg422 residue represents the bottom of the allosteric site.

The ligands were placed in a model of the TP-2 allosteric site comprising 70 atoms, which set the system total to 117 atoms after the addition of the 47 atoms of the 3CJ4 ligand. Section S1 of the Supplementary Materials describes how the number of 70 atoms for the pocket model was decided after a progressive system size assessment (Figure S1) confirmed it was the smallest pocket that maintained the IQA values for the ligand atoms (as shown in Figures S2–S4). The smaller ligand of system 3CJ2 contained 10 fewer atoms, resulting in runs with a 107-atom system (i.e., 107 = 117 − 10). The 70-atom pocket model contains the following: (i) two guanidinium cations, representing the end of the sidechain of the arginine residues (501 and 422), (ii) the sidechain of a methionine (423), (iii) all the atoms from an adjacent histidine (475), and (iv) a serine (476), including the backbone. Note that the arginines and the histidine affect the charge of the system. The model is shown in Figure 2. These residues are conserved among different genotypes of the NS5B protein [38]. Section S2 of the Supplementary Materials lists the coordinates of some of the studied configurations that serve as examples (because listing all coordinates of all configurations would be overwhelming).

**Figure 3:**
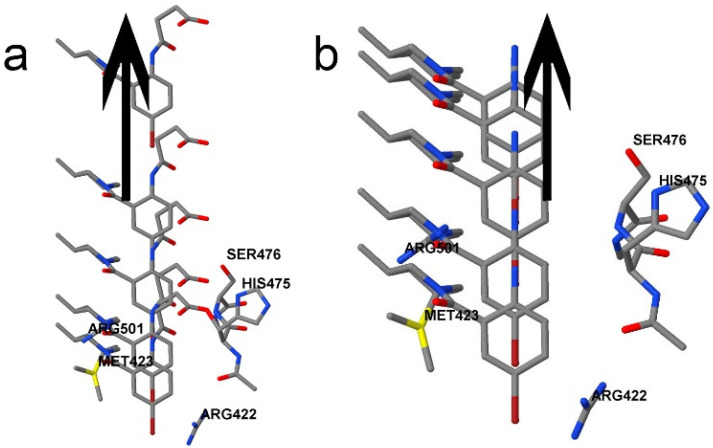
The REG control coordinate. The pocket model is fixed, and different configurations were obtained where the ligand is closer to the pocket than in the PDB file, and others where the ligand is farther than in the PDB file. IQA data are calculated for every atom, and every atomic pairwise interaction, for every configuration. The changes in the IQA energy terms allow highlighting the most important ones. This picture only shows 5 different snapshots along the control coordinate. (**a**) The 3CJ4 ligand contains 117 atoms, and contains an extra succinic acid, which gives it a charge of −1. The charge of the pocket is +3, which creates a total of +2 for the system. (**b**) The 3CJ2 ligand does not contain the negatively charged acid moiety, so the charge of the system remains +3.

The control coordinate in this work was the distance between the ligand and the bottom of the active site. More specifically, it is the distance from the carbon from the succinate chain in 3CJ4 to the NE atom (i.e., N_ε_) of Arg422, which represents the bottom of the pocket. Section S3 of the Supplementary Materials provides a more detailed explanation and a diagram in Figure S5. With the protein pocket atoms fixed, the ligand is moved and different configurations are calculated. This process represents the ligand moving away from the protein pocket representation in a single direction, as Figure 3 exemplifies, with only five configurations for clarity.

## 3. Methodology and Computational Details

### 3.1. Theoretical and Computational Background

#### 3.1.1. Interacting Quantum Atoms

This method has been reviewed many times (e.g., ref. [5]) so we confine this subsection to elementary revision. The IQA partition starts by defining the total energy of a chemical system under the Born–Oppenheimer approximation

(1) $$E_{total}=\frac{1}{2}\sum_{A\neq B}\frac{Z_{A}Z_{B}}{r_{AB}}+\int \hat{h}\rho_{1}(r_{1};r\prime_{1})dr_{1}+\frac{1}{2}\iint \frac{\rho_{2}(r_{1},r_{2}^{})}{r_{12}}dr_{1}dr_{2}$$

where the first term describes internuclear repulsions. The second term, involving the mono-electronic operator and the first-order density matrix *ρ*_1_, can be separated into a kinetic energy term and a nucleus–electron attraction term, while the third term, involving the second-order density matrix *ρ*_2_, describes the electron–electron repulsion. Hence, Equation (1) can be rewritten more simply as

(2) $$E_{total}=V_{nn}+\langle \hat{T}+{\hat{V}}_{ne}\rangle +\langle V_{ee}\rangle$$

The intra-atomic energy (E_self_) and interatomic energy can be extracted from Equation (2), as shown in Equations (3) and (4):

(3) $$E_{self}=\sum_{A}^{}T_{A}+\sum_{A}V_{ne}^{AA}+\sum_{A}V_{ee}^{AA}$$

(4) $$E_{inter}=\sum_{A,B}^{}V_{nn}^{AB}+\sum_{A,B}^{}V_{ne}^{AB}+\sum_{A,B}^{}V_{ne}^{BA}+\sum_{A,B}^{}V_{ee}^{AB}$$

The second-order density matrix can be partitioned into coulombic, exchange, and correlation components (all potential energy terms), which allows us to partition the electron–electron repulsion terms,

(5) $$V_{ee}^{AB}=V_{coul}^{AB}+V_{x}^{AB}+V_{corr}^{AB}=V_{coul}^{AB}+V_{xc}^{AB}$$

The IQA partition groups all intra-atomic terms, in the “self” component described in Equation (3), whereas the interatomic energies are divided into a classical electrostatics term, *V_cl_*, and an exchange–correlation term, *V_xc_*.

Note that the Coulomb term is not analysed as a pure electron–electron energy term but is combined with potential energies involving the nuclei of atoms *A* and *B,* leading to the so-called classical (cl) electrostatic term, denoted *V*_cl_ and defined as

(6) $$V_{\mathrm{cl}}^{AB}=V_{ee,coul}^{AB}+V_{en}^{AB}+V_{ne}^{AB}+V_{nn}^{AB}$$

where $V_{ne}^{AB}$ is the energy associated with the nucleus of atom *A* interacting with the electron density of atom *B*, and $V_{nn}^{AB}$ is the nucleus–nucleus repulsion. The total energy of the system comes from the sum of the three IQA components of all atoms *A* in the system, as can be seen in Equation (7),

(7) $$E_{total}=\sum_{A}E_{IQA}^{A}=\sum_{A}(E_{self}^{A}+\frac{1}{2}\sum_{B\neq A}V_{inter}^{AB})=\sum_{A}\left[E_{self}^{A}+\frac{1}{2}\sum_{B\neq A}\left(V_{xc}^{AB}+V_{cl}^{AB}\right)\right]$$

Finally, there is a practical advantage in *directly* calculating how a given atom *A* interacts with its environment rather than by summing at *A-B* interactions. Pairwise IQA terms (i.e., interatomic energy contributions) for each atom *A* can also be collected into a single term *A-A′*, where *A′* represents [39] all the other atoms in the system except the studied atom *A*. This is not part of the original IQA formulation from 2005 but is implemented in the program AIMAll, which calculates the atomic energies. The calculation of *A-A′* is more accurate; adding all *A-A′* terms to the intra-atomic energies recovers an energy closer to the actual system’s wave function energy than adding the pairwise *A-B* energies. The sum of all *A-B* interactions of an atom also results in the *A-A′* values (minus the integration error) but not as accurately. The many more steps involved in calculating all *A-B* interactions (six-dimensional integrals for each pair of atoms) means a higher integration error and a small discrepancy between the two equivalent values. The *A-A′* terms are computed as one half of the difference of the total energy of an atom *A*, minus the intra-atomic contribution of *A*, or

(8) $$\frac{1}{2}V_{}^{AA\prime }=V_{}^{A}-V_{}^{AA}$$

#### 3.1.2. The Relative Energy Gradient (REG) Method

We use the interacting quantum atoms (IQA) energy partition in conjunction with the relative energy gradient (REG) method [23] to gain chemical insight from the changes in energies that happen as the 3CJ2 ligand and the 3CJ4 ligand move away from the allosteric site. The REG method essentially contrasts the behaviour of the total energy of a system that undergoes a change, with that of the individual IQA energies undergoing the same change. A coordinate of interest is changed, and the IQA energies across the system change as a result. These changes in the IQA energy types highlight the dominant IQA energy contributions in the system at each different configuration. The REG method describes, in a chemical context, which subset of IQA energies behaves as the total system does. REG is implemented in the in-house program ANANKE [23].

Only the essentials of the REG method are described here, while a more thorough definition can be found in the original reference [23]. When a coordinate of interest, i.e., the control coordinate, is changed, then a successive number of configurations of the system is calculated with IQA contributions for each configuration. The sequential changes in IQA energies are analysed, and every IQA term becomes a potential energy surface (PES) whose gradient can be related to the total energy gradient of the system. The maxima and minima in the PES divide the energy landscape into *segments*. Each segment has its own REG values, one for each IQA energy considered.

The total energy and each energetically partitioned term *i* are related using linear regression, as shown in Equation (9),

(9) $$E_{i}(s)=m_{REG,i}\cdot E_{total}(s)+c_{i}$$

where $m_{REG,i}$ is the REG value. The higher the REG value, the more important the corresponding (atomic) energy contribution is in explaining the PES of the total system. Note that there is an equation such as Equation (9) for every energy term *i*. The gradient of each IQA term over a given *segment* is compared with the gradient of the full wave function energy in the segment, as shown in Equation (9). The word *barrier* was used instead of *segment* in the original reference [23]. This work does not involve activation *barriers*, so the word *segment* is preferred. Additionally, the latter term is neutral in terms of the direction in which one travels along the PES; a barrier refers to the uphill direction only. The REG for a given segment of the wave function energy PES can be estimated through least squares linear regression, as shown in Equation (10),

(10) $$m_{REG,i}=\frac{{\left(E_{total}^{translated}\right)}^{\tau }\cdot E_{i}^{translated}}{{\left(E_{total}^{translated}\right)}^{\tau }\cdot E_{total}^{translated}}$$

where ${\left(E_{i}^{translated}\right)}^{\tau }={\left[\begin{array}{cccc} E_{i}(s_{1})-{\bar{E}}_{i} & E_{i}(s_{2})-{\bar{E}}_{i} & \cdots & E_{i}(s_{M})-{\bar{E}}_{i} \end{array}\right]}^{\tau }$ and ${\left(E_{total}^{translated}\right)}^{\tau }={\left[\begin{array}{cccc} E_{total}(s_{1})-{\bar{E}}_{total} & E_{total}(s_{2})-{\bar{E}}_{total} & \cdots & E_{total}(s_{M})-{\bar{E}}_{total} \end{array}\right]}^{\tau }$.

The superscript bar represents an average over *M* data points, one for each configuration, marked by the control coordinate *s*. The translation results from the subtraction of the respective energies. Differentiation with respect to *s* of Equation (9) leads to Equation (11), but in practice, we actually consider finite differences. Equation (11) directly shows the nature of the REG method: the REG value is literally a ratio (i.e., “relative“) of two gradients. In other words, the REG value is a scaling factor relating the gradient of each *i* IQA energy term to the gradient of the total system (for that segment),

(11) $$\frac{dE_{i}(s)}{ds}=m_{REG,i}\frac{dE_{total}(s)}{ds}$$

Note that when *s* is a cartesian coordinate, the REG value becomes a ratio of forces. The method requires the full set of IQA data (*A-A′* and *A-B*) of several configurations, with the coordinate of interest *s* sequentially changing across systems.

The REG value is positive if the energy contribution that it describes is contributing towards the general direction of the gradient of *E_total_*. It is negative if the changes in the segment for the given energy are opposite to the overall behaviour in that segment. A positive REG value has the same sign in energy gradient as the total system in that segment. This allows for a ranking of REG values, and the largest changes will be the most significant, in either direction. A different set of REG values are calculated for each segment. It is important to remember that it is unlikely that a single set of IQA terms will represent the overall PES behaviour of the system for all configurations. Each segment will have its own ranking of terms whose changes resemble those of the whole system PES in that segment.

The IQA partition can be generalised by grouping atoms into fragments [40], which allows the analysis of whole-group interactions, in an approach referred to as the Interacting Quantum Fragment analysis (IQF) [41,42,43]. The IQA formulation of Equations (1)–(8) is maintained, but *A* and *B* now indicate fragments instead of atoms. This approach is used to obtain insight about the changing interactions between the pocket and ligand as the ligand exits the allosteric site.

### 3.2. Computational Details

The increase in computational resources has allowed QCT to extend beyond its original action radius of dozens of atoms to that of hundreds of atoms. The fact that systems with more atoms can now be analysed through the QCT framework means that biological systems can be looked at in a more realistic way, closer to their in vivo state. The recent advances in the program AIMAll [44] allow for IQA energies to be calculated for just over 100 atoms for the bulk of the current work, with a maximum of up to 400 according to our experience. The total size of the systems in which IQA can be performed is only limited by the computational power available. For example, a carbon atom in an acetate moiety of a ligand takes about 10 h to be calculated in a 4-core node for a 117-atom system. A roughly equivalent carbon atom from an acetate moiety in a ligand in a 367-atom system takes about 61 h on a similar node. The calculation times for the roughly equivalent atoms take six times as long when the number of atoms in the system is three times larger.

If the full IQA partition of a configuration could not be obtained within a week on a 4-core node, the whole configuration was discarded. Hence, due this constraint of a week on the University’s Computational Shared Facility (CSF) there is an unequal number of configurations between systems. The complete IQA partition from 17 configurations was obtained for the 3CJ4 system, while 22 were obtained for the 3CJ2 system. All ab initio calculations were performed using GAUSSIAN09 [45] at the B3LYP/6-31+G(d,p) level of theory. The IQA calculations were performed by AIMAll16, turning off the TwoE [46] option, which was used by default by AIMAll in the version used (it has since been removed from default settings). The extension of IQA that allows AIMAll to work with the functional B3LYP has been shown to give chemically meaningful results [39]. The 6-31+G(d,p) basis set has been benchmarked as a good trade-off between computational cost and accuracy [47].

## 4. Results

### 4.1. The PES of the Total System

**Figure 4:**
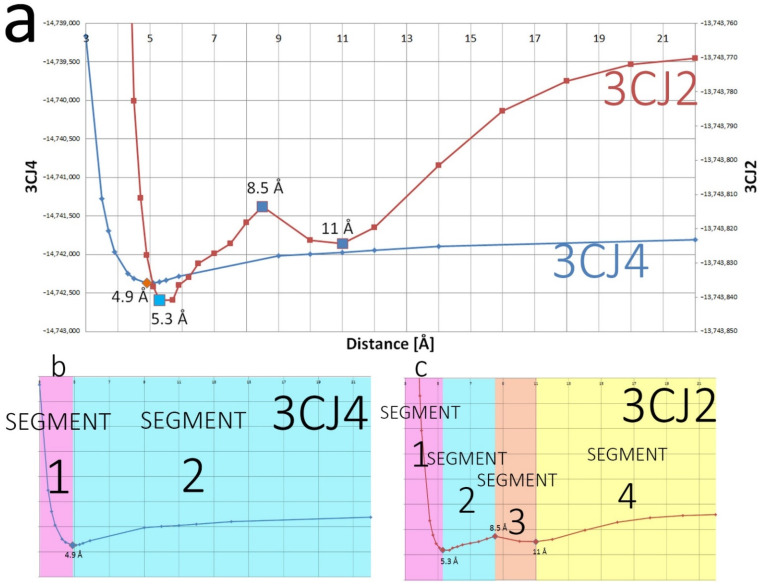
(**a**) The full wave function energy PES for both systems as the ligands move away from the pocket. The energies for the blue 3CJ4 system are far more negative than those for the red 3CJ2 system, simply because 3CJ4 has more atoms. The global minimum is slightly shifted but the shape of the curves is similar. Both curves have been scaled to the 22 Å progression over the x axis. (**b**) The 3CJ4 wave function energy graph shows the energy decreasing as the ligand moves away from the pocket until the single global minimum, the optimal distance between the drug candidate and the pocket, and afterwards, energy becomes less negative. (**c**) The 3CJ2 system has a similar behaviour to 3CJ4 but introduces a drop in energy between 8.5 Å and 11 Å. As a result, 3CJ4 has 4 segments compared with only 2 for 3CJ4. All interactions present in 3CJ2 are also present in 3CJ4 but the graph in (**b**) demonstrates that some interactions in 3CJ4 mask the changes in 3CJ2, namely the hydrogen bonds that are present only in the 3CJ4 system.

The PES formed by the wave function energies shows an attractive interaction at long range and a repulsive one at short range, akin to a Morse potential. Figure 4 shows that the system 3CJ2 (red line) exhibits local extrema, a feature that is absent in the system with ligand 3CJ4 (blue line). The distance at which the global minimum occurs is shifted between the two systems. The 3CJ2 ligand forms only one hydrogen bond with Arg501, while the 3CJ4 ligand forms four, that is, the same hydrogen bond with Arg501 but with three extra hydrogen bonds: with the Ser476 backbone, as well as with the Ser476 and His475 sidechains. In the 3CJ2 system, the global minimum simply corresponds to the optimal ligand–pocket distance for the single hydrogen bond of the system. However, in 3CJ4 the number of hydrogen bonds increases to four; hence, the global minimum occurs at a distance that is favourable for all four hydrogen bonds at the same time. In its global minimum, the 3CJ4 ligand is 0.4 Å closer to the pocket than in the optimal conformation for its parent 3CJ2, as can be seen in Figure 4a.

### 4.2. Relative Energy Gradient (REG) Method

#### 4.2.1. Generalities

The IQA partition (*A-A′* energies) accounts for virtually all of the wave function energy, with less than 0.001% of the energy lost due to a negligible integration error. The REG method was performed on the full set of IQA data, allowing for two rankings of the IQA terms. Firstly, one ranking involving the atomic energy contributions as a whole or “*A-A′* interactions”, which means a ranking of the 117 atoms for the 3CJ4 system (and 107 atoms for 3CJ2). This ranking does not specify which IQA component (out of *E_self_, V_xc_*, or *V_cl_*) dominates the changes. Secondly, a ranking of the 13,689 pairwise terms for 3CJ4 (and 11,449 for 3CJ2) or “*A-B* interactions”, which allows us to know which types of IQA energies are the most important and, if it is a pairwise interaction, which two atoms are responsible for it. The IQA terms with the largest positive REG values dominate the behaviour of the total system for that segment, while the largest negative REG values are the IQA terms whose changes work against the system’s behaviour. Most of the IQA terms experience negligible changes and thus have REG values close to zero. Only selected rankings are reported here, with the majority of *A-B* tables for 3CJ2 present in the Supplementary Materials.

#### 4.2.2. The 3CJ4 System

The first segment of 3CJ4 describes a sharp decrease in energy as the distance increases from very small values to the global minimum (see Figure 4a). The energy of the system becomes less negative when the ligand and pocket are too close, and becomes more favourable (i.e., negative) as they separate towards the distances of the global minimum. In the initial configurations, the atoms are shown to be so close that the program JMOL [48] depicts them as covalently bonded (shown in Figure S6). These same atoms obtain a more negative IQA energy as distance increases towards the global minimum, located at 4.9 Å.

Table 2 shows the most positive REG values for full atomic *A-A′* interactions in segment 1, showing the importance of atoms such as H91 (His475), S69 (Met423), and O47 (ligand, acid moiety). Because their REG values are positive, they support the energy profile of the total system and thus favour the ligand and pocket separating. In other words, they add to the repulsive character of this segment. Put differently again, they are the atoms that are affected the most by compression when the ligand and pocket are too close to each other.

Table 3, on the other hand, shows a ranking for the full *n^2^* (where *n* is the number of atoms) IQA energy terms, which include both *E_self_* terms (*n*) and the pairwise contributions of *V_xc_* and *V_cl_* (twice *n(n-1)/2*). In this table, three out of the top five IQA terms in favour of the ligand–pocket separation are steric terms. This shows that sterics are the driving force for segment 1, at short range. They contribute to the energy becoming more negative as the distance increases towards the global minimum, and correspond to atoms that were compressed in the configurations that form segment 1. Table 3 also shows that some atoms (H91 and O47) are involved in interactions in both senses, as they appear both at the top and bottom: a given atom can have pairwise contributions both in favour of and against the system’s behaviour, depending on which other atom it interacts with. The top pairwise terms in Table 3 involve H91, the main atom in favour of the system‘s de-compression (as shown in Table 2). This H91 atom in His475 is not an atom that is involved in any strong electrostatic interactions beyond segment 1, once the ligand–pocket compression is past its highest point. Note as well that the H91–O47 interaction is quite relevant at first, but loses any importance once we are out of segment 1. The oxygen atoms from the ligand acid moiety (O46 and O47) are so close to H91 in His475 that even covalent interactions appear between them. However, the sum of all interactions involving both O47 and H91 results in both atoms being in favour of the ligand and pocket separating, as shown in Table 2. This O47–H91 pair attraction is but one among a sea of different interactions occurring at the same time. Hence, looking at the *A-B* ranking only may give a wrong first impression.

We now move onto the other segment in the 3CJ4 system: segment 2 describes the full wave function energy becoming less negative as the distance between the pocket and the ligand increases, away from the global minimum. However, it is more intuitive to describe segment 2 in the opposite direction: as the distance decreases from very large to that of the global minimum, the energy becomes more negative. Segment 2 is then seen as an attractive process, and the question then is what brings ligand and pocket together. Table 4 shows the top and bottom energy terms for the *A-A′* ranking (full atomic contributions) for segment 2. Note that the REG values are intrinsic. This means that their signs are independent of the direction of travel along the segment. In other words, the signs express an intrinsic relationship between the behaviour of the total system and that of an individual IQA energy term.

**Figure 5:**
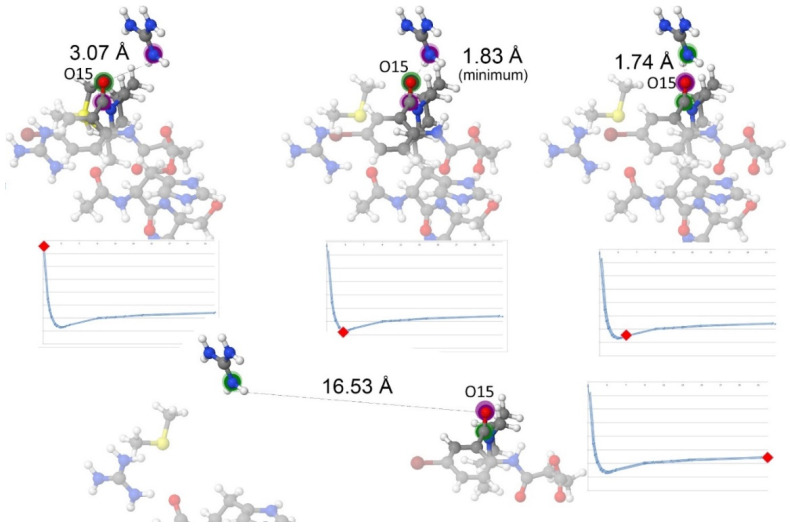
The so-called neighbour effect illustrated for four configurations, each marked by an inset representing the full wave function energy profile (blue) with a red diamond marking that particular configuration. The atoms involved in hydrogen bonds, namely the hydrogen donor (N48 in Arg501) and hydrogen acceptor (O15 in the amide of the ligand), show opposite behaviours to each other. The opposite behaviour between the hydrogen donor and the hydrogen acceptor is maintained once past the global minimum and entering segment 2. After transitioning the segment, the behaviour of these two atoms remains opposite to each other, but they now contribute to the whole system in a switched sense, as marked by the coloured circles shown surrounding the atoms. The green circles surrounding an atom represent increased stability (a more negative energy), while the purple circles represent decreasing stability (a less negative energy). We travel along the energy profile from left to right (i.e., the ligand moves away from the pocket). Initially, the hydrogen acceptor O15 is shown surrounded by a green circle, as its energy becomes more negative until the global minimum. When the distance increases further, away from the global minimum, segment 2 starts and the energy of O15 becomes less negative as the distance increases. Meanwhile, the hydrogen donor (N48 in Arg501) and the neighbour (carbon of the amide of the ligand) of the hydrogen acceptor show opposite behaviour. The hydrogen atom itself from the hydrogen bond can make a contribution in both senses depending on the case, although the REG value will be small and often not rank as an important contributor.

Overall, the REG values are larger than the ones calculated for segment 1. Immediately, it is noticeable that the atoms with the top REG values are all hydrogen acceptors in the four hydrogen bonds present. Because their REG values are positive, these four atoms (O47, O46, O15, and O38) have energies that become more negative as distance decreases. As a result, they follow (i.e., support) the energy profile of the total system. Because of their large absolute value, these REG values are prominent in explaining what brings ligand and pocket together. At the bottom part of Table 4, there are three carbons (C14, C10, and C45) that show the largest negative REG values in terms of absolute value. Each of these carbons are covalently bonded neighbours to the top positive atoms (see the hydrogen acceptor atoms mentioned above): C14 for O15, C10 for O38, and C45 for both O46 and O47. So, the atoms (carbons) that contribute most *against* the system’s behaviour are covalently bonded to the atoms (oxygens) that contribute most *in support* of the system’s behaviour. This “neighbour effect” was observed thanks to the REG method. Stated in simple terms: *a carbon atom would rather not have its covalent neighbour oxygen form a hydrogen bond*. Similar results were also observed by Vallejo Narváez et al. in dimers of amides and imides [49]. This effect was observed for all the hydrogen bonds present in both 3CJ4 and 3CJ2. The hydrogen atoms themselves in the hydrogen bonds do not show a clear trend. Figure 5 shows a diagram of the observed “neighbour effect”.

We now move on with an analysis of the interatomic interactions. The ranking of the REG values of *A-B* pairwise interactions can give more insight into which type of IQA energy is responsible for the neighbour effect. In other words, the *A-A′* ranking of Table 4 gives information about which atoms best explain the system’s behaviour, but not the types of IQA energy that made these atoms do so. Table 5 shows the top and bottom IQA terms of a pairwise *A-B* ranking. In all cases, *V_cl_* are the main contributions. The acid moiety of the ligand makes the most important interactions both in favour of and against the system’s behaviour. Furthermore, the pairwise interactions arising from the hydrogen bond of O15-Arg501 appear as important contributors in favour of the system’s behaviour. That particular interaction is noteworthy because it is an interaction that later remains for 3CJ2, and in that system it becomes the most important contribution, as shown in Tables S5 and S7. Table 5 shows an important difference from Table 3, as Table 5 confirms that the *E_self_* energies are no longer the terms that best describe the system’s energy profile in this segment 2. No *E_self_* or *V_xc_* term appears as a top contributor, neither in favour of nor against the system’s behaviour. The top interaction in favour of the system’s behaviour is the interaction between C45 and N93, which have opposite partial charges and show an attraction at the global minimum. C45 is covalently bonded to two oxygen atoms (both hydrogen bond acceptors) and acquires a partial positive charge, whereas N93 is the hydrogen donor of one of the hydrogen bonds and acquires a partial negative charge. Although their opposite charges cause them to have an attraction with each other, this should not distract from the fact that both atoms appear as top contributors against the system’s behaviour for segment 2, as shown at the bottom of Table 4. This means that regardless of there being a strong attractive interaction between the two atoms at the global minimum, this is but one among a sea of interactions and overall their stability increases as the ligand and pocket separate. The highlighted contributions in Table 5 represent the hydrogen acceptor and the hydrogen atoms for one of the hydrogen bonds in the system.

#### 4.2.3. The 3CJ2 System

The 3CJ2 system contains four segments. Segment 1 is equivalent to segment 1 in 3CJ4. In both segments, the system energy becomes more negative as the ligand and pocket separate from each other until reaching the global minimum. Segments 2 and 4 in 3CJ2 are equivalent to segment 2 in 3CJ4. In those segments, the system energy becomes less negative as distance increases beyond the global minimum. Segment 3 is unique to 3CJ2, and the system’s energy becomes more negative for a short distance due to the interactions of the bromine atom in the ligand. Those interactions also happen in 3CJ4 but their importance is surpassed by the other stronger interactions, namely all the other hydrogen bonds in that system. In summary, the information regarding segments 1, 2, and 4 are shown here for the sake of completeness while segment 3 deserves special attention.

In 3CJ2, the ligand does not have the acid moiety so there is only one hydrogen bond, which involves the oxygen atom from the tertiary amide that links the piperidine and bromo-aryl moieties of the ligand, that is, atom O15 and residue Arg501. As was pointed out by the REG method in 3CJ4 (*A-A′* ranking, Table 4), the hydrogen acceptors are the atoms whose energy gradient behaves the most as the energy gradient of the total system, in the segments where the protein and the ligand are farther than in the global minimum and the system’s behaviour is for the energy to become less negative. Nevertheless, three out of the four hydrogen bonds present in 3CJ4 are not present in 3CJ2, which means that the O15-Arg501 hydrogen bond interaction acquires dominance. At short range, when the ligand and pocket are closer together than at the global minimum, the steric intra-atomic energies of compressed atoms are the main drivers, because the ligand and pocket atoms become too close to each other. This is the case too for 3CJ2. Residues His475 and Ser476, largely important in the 3CJ4 system (because of compression in segment 1 and because of the hydrogen bonding disruption in segment 2), stop representing the system behaviour and no longer have a large REG value. There is no acid moiety in the ligand anymore; His475 and Ser476 are not compressed by any moiety when the ligand is close, and they do not form hydrogen bonds. Thus, no His475 or Ser476 atoms appear in the top terms of the REGs in any segment that is equivalent to those in the 3CJ4 system.

The *A-A′* ranking for segment 1 of 3CJ2 (shown in Table S4) is closely similar to that presented in Table 3, with the difference that the atoms in the acid moiety are not present. Thus, those atoms and the corresponding pocket atoms that were compressed by them in 3CJ4 do not appear. The top atom in Table 3, H91, labelled H81 in 3CJ2 (3CJ2 is 10 atoms smaller), no longer appears in the top atoms of the ranking. The acid moiety, which compressed this atom in segment 1 of 3CJ4, is gone. Table S5, on the other hand, shows the *A-B* ranking for the same segment 1 of 3CJ2, allowing us to see the type of IQA energy. The *A-B* ranking allows one to see that once again it is the steric energies *E_self_* that best represent the energy behaviour of the full system in segment 1. The top energy terms in favour of the system’s behaviour are all atoms involved with the compression of Met423 and the 3CJ2 ligand, namely atoms from the residue such as the sulphur atom, CE (i.e., C60 in Met423, see Table S5) and one of the hydrogen atoms that cap it, as well as ligand atoms. Even the *A-B* terms that are against the system’s behaviour, at the bottom of Table S5, are related to the same compression. However, they are exchange–correlation and classical electrostatic energies that benefit from the short distance between pocket and ligand (further supported by the fact that most are *V_xc_* energies). When the ligand and pocket are close, inevitably a handful of IQA terms will have an attractive contribution. These bottom pairwise terms of Table S5 show an energy profile that goes against that of the system while approaching the global minimum (the system’s energetic profile shows that the energy becomes more negative as the global minimum is approached, but for these bottom terms of Table S5 the energy becomes less negative as the global minimum is approached). Although the bottom terms of Table S5 also involve atoms from both Met265 and the ligand, these are only a handful of interactions against the system’s behaviour. The majority of interactions benefit (i.e., acquire a more negative energy value) from the ligand and pocket separating. In other words, the fact that some pairwise interactions that involve, for example, the sulphur atom of Met423, show an energy profile against that of the whole system in segment 1 in Table S5, is just a small part of the story, as the *A-A′* ranking had already confirmed that the sulphur atom actually behaves in favour of the system energy’s profile for that segment (Table S4). Furthermore, interactions involving the same sulphur atom are also present in Table S5, in favour of the system’s behaviour.

Segments 2 and 4 of 3CJ2 are shown together in Table 6 because they are similar to each other. In 3CJ4, segments 2 and 4 are one and the same. However, for 3CJ2 the interactions of the bromine, at the bottom of the ligand as it exits the allosteric site, acquire a preponderance that they do not have in 3CJ4, and split the segment. Segment 2 in 3CJ2 starts at the global minimum at 5.3 Å and continues until 8.5 Å. Segment 4 encompasses the distances from 11 Å onwards. At the global minimum, the lone hydrogen bond of 3CJ2 (between Arg501 and the O15 from the tertiary amide in the ligand) is in an optimal position. The global minimum is at a different position than the global minimum for the 3CJ4 system. In 3CJ4 the simultaneity of the hydrogen bonds means that the global minimum occurs at the point where the energy is the lowest, corresponding to a position that accommodates all hydrogen bonds on average. The global minimum in 3CJ2 occurs at a distance that is optimal for the single hydrogen bond in the system. The top atoms in the *A-A′* REG ranking for segments 2 and 4 are shown in Table 6. The importance of O15 and its covalently bonded neighbour, C14, is demonstrated (which are the hydrogen acceptor and its neighbour, respectively), as those two atoms are the main contributions in favour of and against the system’s behaviour, respectively. The neighbour of the hydrogen acceptor of the hydrogen bond, C14 in this case, acquires a more negative energy once the distance for the hydrogen bond is no longer optimal. As a result, C14 goes against the system’s behaviour, which is more evidence for the neighbour effect that was mentioned before.

An atom that appears as the second top contributor against the system’s behaviour for segment 4 but not for segment 2 is the bromine at the bottom of the ligand. As will be reviewed shortly, the interactions of this bromine atom create segment 3. These interactions mean that the bromine atom acquires a more negative energy, not only for the duration of segment 3 but in distances past it. The overall energy of the system creates a local minimum (as shown in Figure 4) and recovers the system’s behaviour for segment 2 once the bromine interactions are not enough to keep the overall energy from the whole system to continue becoming more negative with increasing distance. The *A-B* ranking for the segment 2 of 3CJ2 is in Table S6, whereas the *A-B* ranking for the highly similar segment 4 can be found in Table S7. Further analysis for segments 2 and 4 of 3CJ2 can be found in the Supplementary Materials.

Segment 3, which is unique for 3CJ2, arises because of bromine interactions, which were masked before (in segment 2 of 3CJ4) by the stronger interactions of hydrogen-bonded atoms. The bromine interactions become the most important terms in the distance range from 8.5 to 11 Å, and show a suitably high REG value in the ranking. Table 7 shows the full atom *A-A′* ranking of REG values in segment 3. The atoms involved in the hydrogen bond (C14 and O15) have absolute REG values greater than that of bromine, and are thus still ranked as more important than the bromine in the *A-A′* ranking. However, they do not explain the cause of segment 3. They just show a contribution with opposite sense with respect to their own contributions in segments 2 and 4 because the system’s energy profile has changed due to a local maximum, but they are not involved in creating this local maximum. The energy of the hydrogen acceptor (O15) keeps becoming more negative as distance decreases towards the global minimum. The only atom that appears in the top terms of Table 7 and that did not appear in segment 2 of 3CJ4 is the bromine in the bromo-aryl moiety of the ligand, which reveals unprecedented importance in an effect that was also present in 3CJ4, albeit masked.

The top pairwise interactions from an *A-B* ranking of segment 3, reported in Table S8, also do not explain the importance of the bromine. All the top terms are related to the hydrogen bond and the neighbour effect, particularly electrostatic *V_cl_* interactions. The local maximum and minimum that create segment 3 actually happen because of the interactions of atoms not involved in the hydrogen bond, particularly those of the bromine of the bromo-aryl moiety, Br1. However, not a single pairwise term that involves the bromine appears in the top pairwise interactions shown in Table S8; the atoms related to the hydrogen bond dominate the first places in the ranking. Another ranking was carried out, this time only considering the pairwise *A-B* interactions of bromine, shown in Table 8. They show an attractive electrostatic interaction between the partially negative bromine and the partially positive carbon atoms of certain residues, which are responsible for the sudden increase in stability and the creation of segment 3.

**Figure 6:**
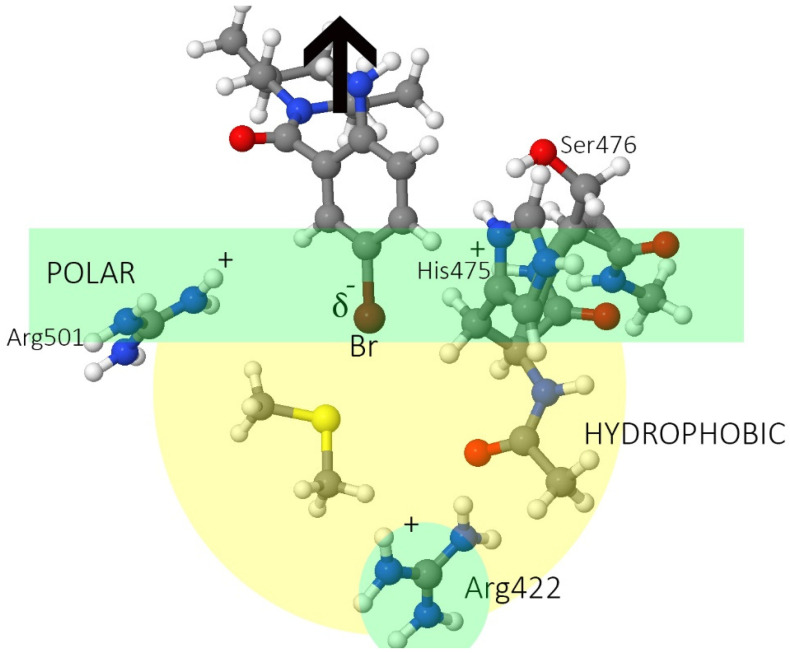
Representation of the charges in the allosteric site as the 3CJ2 ligand moves away (upwards), showing how the bromine from the bromo-aryl moiety affects the energy landscape. As the ligand moves away from the pocket, the bromine interacts with the positive charges in the outer part of the pocket, leading to a small segment where the system’s energy becomes more negative as the ligand moves away, which explains segment 3.

**Figure 7:**
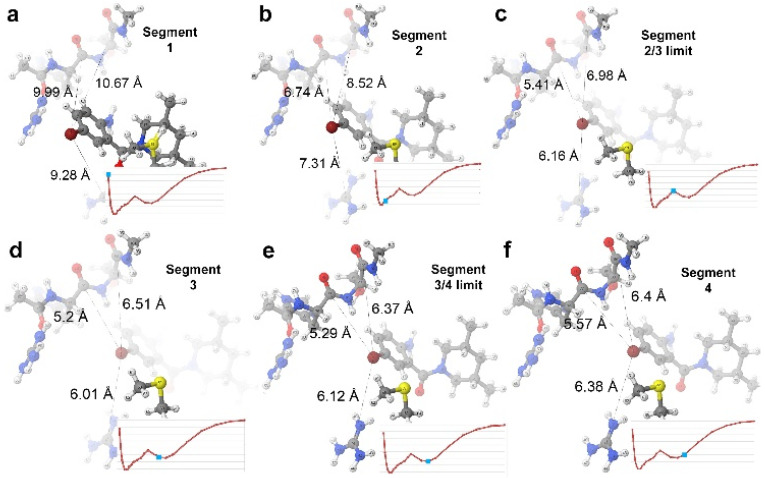
Geometries associated with key points (blue) on the full system’s PES (red curves) of 3CJ2 as its ligand moves away from panel (**a**–**f**) crossing all four segments. The local maximum and local minimum are explained by the interactions of the partial negative charge of bromine (brown atom, of the bromo-aryl moiety of the ligand) with the positive and negative partial charges of the outer parts of the allosteric site representation as the bromine atom closes in and departs from the residues in the outer parts of the allosteric site. The bromine gains importance from segment 3 onwards, starting in panel (**c**), corresponding to the local maximum. The local maximum, panel (**c**), and local minimum, panel (**e**), appear because the negative bromine starts interacting electrostatically with the mainly positive partial charges of the outer parts of the site, which were absent in the hydrophobic cavity. Through all the panels of this figure, we show the progression of distance values between bromine and the three atoms (in residues His475, Arg501, and Ser476, as shown in Table 8) that form the top pairwise interactions with bromine. We also show these distances to bromine change as the ligand moves away from the pocket (and are at their shortest during segment 3). The local minimum appears when the distance between bromine and these atoms is minimal. This effect appears in both 3CJ4 and 3CJ2 but the effect is masked in the 3CJ4 ranking. The many more hydrogen bonds between the pocket and the 3CJ4 ligand cause the involved atoms to have a much higher REG value than bromine, despite the bromine experiencing the same interactions across both systems.

Figure 6 shows a diagram of the charges experienced by the bromine across the allosteric site, while Figure 7 shows the changing distances from bromine to its top interacting atoms (from Table 8, the *A-B* ranking) and how these distances become minimal in segment 3. The *E_self_* of bromine is also an important term, gaining stability when the bromo-aryl moiety is not in the allosteric site anymore. This effect starts at 8.5 Å and is not an important contributor when the ligand is closer to the pocket, despite the bottom of the allosteric site having a positively charged guanidinium group from Arg422 [50]. This suggests that, at the global minimum, the Arg422 and the bromine are close enough to interact electrostatically but not enough to compromise its sterics too much (when the ligand is in the allosteric site, the volume of the bromine atom decreases to 90% of its volume compared with when the ligand is free). However, the bromine’s intra-atomic energy benefits from the space gained when free from the allosteric site. The *E_self_* energy of the bromine becomes more negative when the bromine is out of the allosteric site. This means that the sterics of the bromine atom are affected when the ligand enters the allosteric site, but the interactions of the rest of the ligand compensate for this.

### 4.3. Interacting Quantum Fragments Analysis

**Figure 8:**
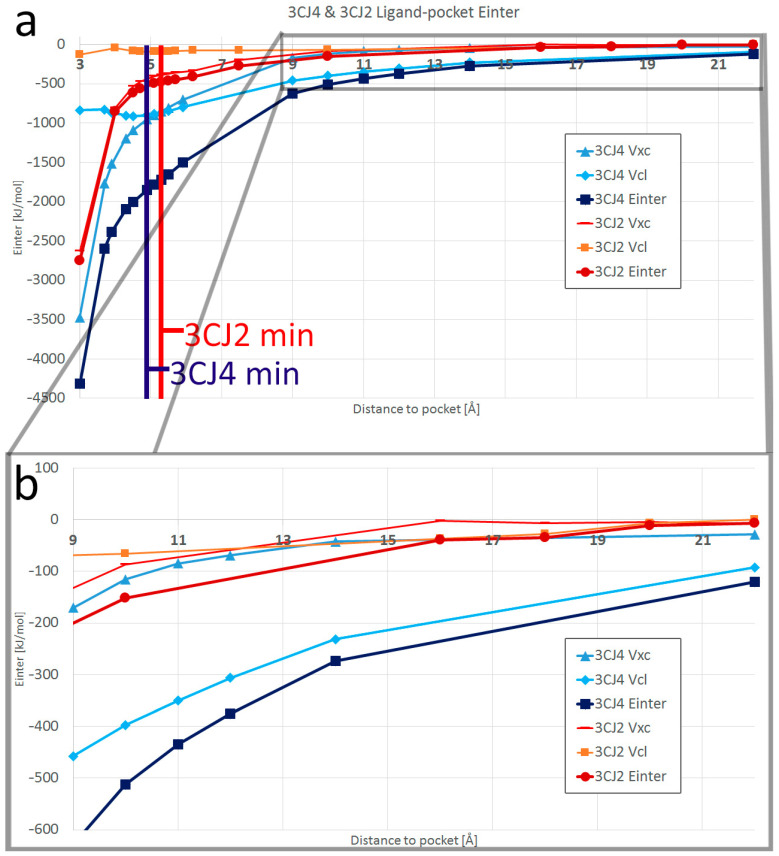
*E_inter_* progression between the ligand and pocket of the studied systems using interacting quantum fragments (IQF). In the top graph (**a**) the full progression is shown, and at short range, the V*_xc_* component dominates the interaction. This preponderance lasts practically until the distance reaches the global minimum for the more polar 3CJ4 system, but in the case of 3CJ2, the interaction has a mainly covalent character for longer, until a separation of 10.5 Å, well into the distances of segment 3. The global minimum distances for both systems are shown as vertical lines (shades of blue for 3CJ4 and shades of red for 3CJ2). In (**b**), an enlarged version of the interactions at the longer distances between the ligand and the pocket indicate an increased relevance of the classical electrostatics component of the interaction (*V_cl_*).

We applied IQF to each of the NS5B system configurations, where one fragment is the ligand and the other fragment the pocket. For each configuration, the ligand–pocket interaction energy was obtained, along with its separation in *V_xc_* and *V_cl_*. The self-energies from each fragment were obtained as well in order to analyse deformation energies. In the interaction energy progression plots, shown in Figure 8, it is immediately noticeable how the ligand–pocket interactions are strongest at short range. Indeed, the REG method has shown that the steric repulsion is the main energy component of the total system at short range, but this effect cannot be seen when analysing E*_inter_* with IQF, as the steric repulsion between the fragments is not considered for the ligand–pocket fragment interaction calculation. However, the steric repulsion can be seen in the high deformation energies of the ligands in the closest configurations, as shown in Figure S7. The interaction energies at the shortest distances show a strong attraction. The V*_xc_* component dominates at first, its preponderance waning as distance increases. At longer distances, the electrostatics become the dominant component of the interaction in both systems. The rate of the decrease in V*_xc_* preponderance was much slower in 3CJ2, where there are fewer atoms that form hydrogen bonds. One must keep in mind that the interaction energy, as a whole, quickly becomes less negative at longer distances, as can be seen in Figure 8. Comparing the E*_inter_* values with those of the global minimum configurations allowed us to obtain the changes as percentages, which we report next.

Figure 8 shows both the 3CJ4 and 3CJ2 ligand–pocket interaction energy progression, where the V*_xc_* contribution shares the shape with the total E*_inter_* at short range. In 3CJ4, the interaction between ligand and pocket begins at 3 Å with 80% covalent character. This percentage becomes smaller as the distance increases, and crosses 50% at a separation of 5.3 Å, slightly after the global minimum at 4.9 Å. Meanwhile, the 3CJ2 ligand–pocket interaction, having fewer polar interactions, begins with an interaction energy that is 95% covalent at 3 Å. The contribution from V*_xc_* also decreases with distance, but the threshold of 50% is reached at the longer distance of 11 Å, which coincidentally (and interestingly) marked the end of segment 3.

Figure S8 compares the magnitude of the 3CJ4 ligand–pocket interaction energy with the magnitude of the 3CJ2 ligand–pocket interaction energy. As 3CJ4 contains an extra moiety and three extra hydrogen bonds, a more negative interaction energy was expected for all the studied configurations. However, a comparison whenever there are equivalent frames between the system showed that this proportion is not constant. At short range, the 3CJ4 ligand–pocket interaction energy is only 1.7 times that of 3CJ2. This factor increases to about 3.5 times the ligand–pocket interaction of 3CJ2, while the ligand and the pocket have an increasing separation distance from 5 to 15 Å.

**Figure 9:**
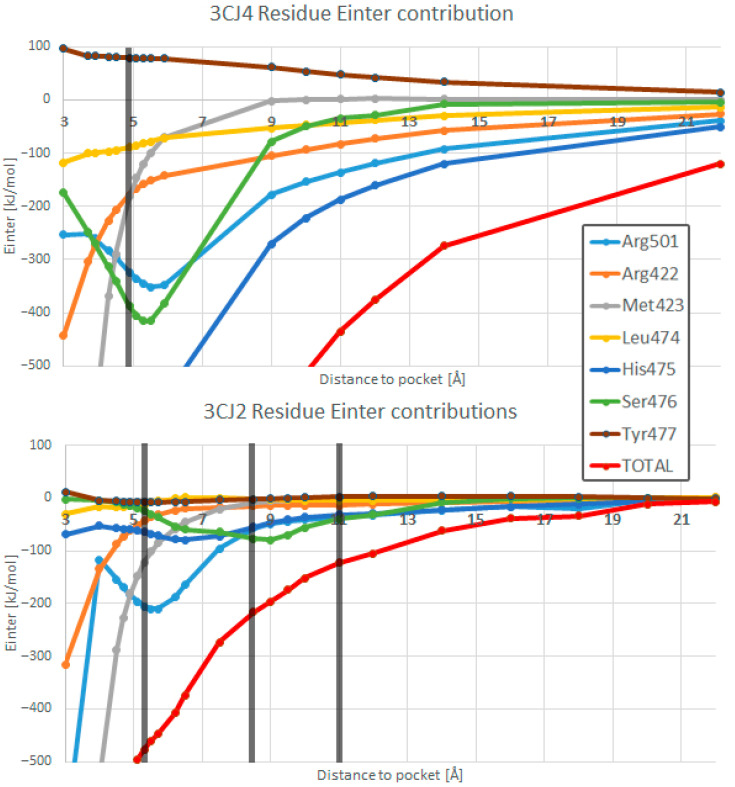
Contributions to ligand–pocket E*_inter_* for each of the seven residues in the pocket: (top) 3CJ4 and (bottom) 3CJ2. Vertical black lines represent the separation between the segments studied with the REG method: 2 segments for 3CJ4 and 4 segments for 3CJ2. In segment 1 for both systems, the strongest contributor is Met423, but its contribution wanes as the distance increases. Arg501 is the top contributor in segment 2 of 3CJ2 (responsible for the only hydrogen bond) but its contribution in 3CJ4, although important, is eclipsed by the other residues forming hydrogen bonds with 3CJ4 (His475 and Ser476). Residues His475 and Ser476 only have an important contribution when the extra chain of ligand 3CJ4 is present.

The group analysis also allows us to determine which residues are the ones that contribute most to the ligand–pocket binding. It was expected for the dominating residues to change depending on the segment of the PES. The two graphs in Figure 9 show the contribution for E*_inter_* from all seven residues in the pocket (five main ones, Arg501, His475, Ser476, Arg422, and Met423, and a handful of atoms from Leu474 and Tyr477), with vertical lines separating the segments. In segment 1, Met423 and His475 are the residues with the strongest interaction for 3CJ4, but for 3CJ2 only Met423 retains this feature because of the absence of the extra chain. Once distance increases to segment 2 in 3CJ4, the main residues are the three that form hydrogen bonds: His475, Ser476, and Arg501. Ser476 is the residue whose contribution wanes first. Regarding segment 2 for 3CJ2, Arg501 is the most negative contribution, as it forms the lone hydrogen bond of the system. However, when reaching segment 3, the interactions are dominated by Ser476. Two atoms (N and H) in the backbone interact covalently with a close part (1.6 Å) of the aryl moiety of the 3CJ2 ligand during segment 3. In 3CJ2′s segment 4, the contribution of Ser476 quickly wanes as the aryl moves away. Instead, Arg501 and His475 become the main contributions of a minimal interaction. The two graphs in Figure 9 use the same scale to show the difference in E*_inter_*.

**Figure 10:**
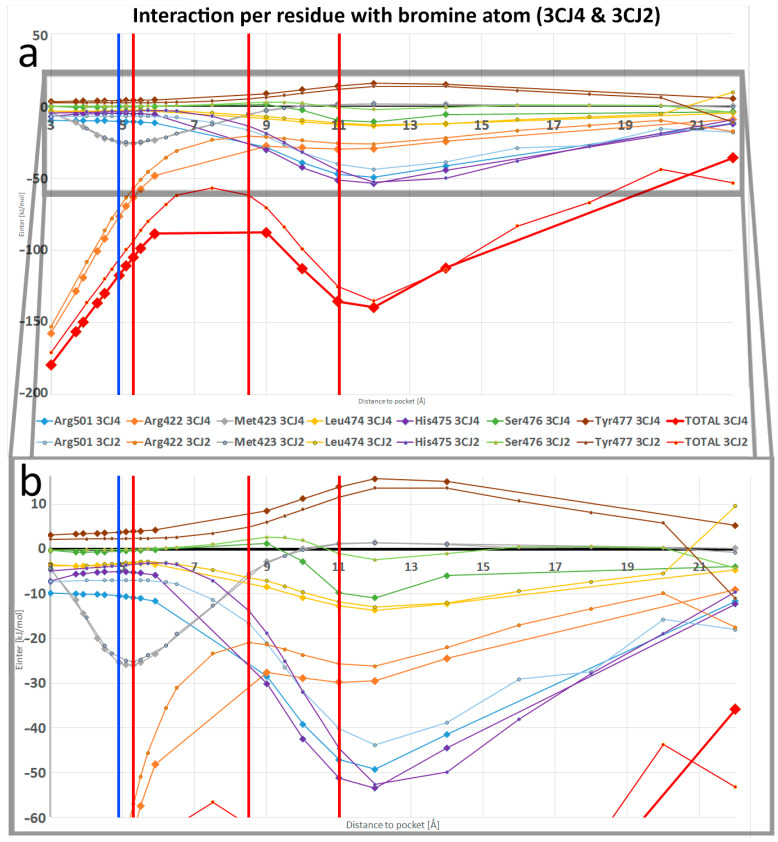
Contributions of each residue in 3CJ4 and 3CJ2 to the pocket–bromine interaction. Each contribution to the interaction is shown next to another interaction of the same colour, that is, the same equivalent interaction in the other system. All curves retain their shape regardless of the system. The separation between segments of the system’s PES is shown as vertical lines in blue (3CJ4) and in red (3CJ2). Panel (**a**) shows that even the total pocket–bromine interaction energy profiles are similar in both magnitude and shape. Panel (**b**) is an enlarged version of panel (**a**), which focuses more on the contributions per residue and confirms that the interactions of bromine in segment 3 of 3CJ2 are largely caused by His475, Arg501, and to a smaller degree by Ser476. This insight had also been obtained with the REG method (Table 8). These interactions in 3CJ2 are the same as well for bromine in 3CJ4, but, as shown in the PES of Figure 4a, in 3CJ4 there is no local maximum and minimum caused by the bromine interactions; its effect is masked in the 3CJ4 PES by all the extra hydrogen bonds.

The local maximum and local minimum in the 3CJ2 PES that create segment 3 in Figure 4 do not have an effect on the ligand–pocket interaction energy progression shown in Figure 8, because the interaction continuously becomes less negative as distance increases. However, we know from the REG method that the bromine atom in the ligands was responsible for the local maximum and minimum in the 3CJ2 PES, so we used IQF to analyse the pocket–ligand binding contributions of that atom in particular. We had no reason to believe that the binding contributions from bromine were any different between the 3CJ4 and 3CJ2 systems. The IQF analysis confirms that the binding contributions of the ligand bromine atom to the pocket are highly similar across the two systems (as shown in Figure 10). This means that the interactions of bromine are maintained after improving the drug candidate, which is precisely the basis of the fragment-based drug design method through which the 3CJ2 and 3CJ4 candidates were designed. The contributions from bromine are the same but can only be seen in the full PES of 3CJ2 because in 3CJ4 the hydrogen bonds between the succinic acid chain and residues His475 and Ser476 mask the bromine effects in the PES.

In both systems’ segment 1, Arg422 is the main residue interacting with Br1, which was expected, as it is the residue at the bottom of the allosteric site. This Br–Arg422 interaction has, at the shortest distance, an 84% and 86% covalent component for 3CJ4 and 3CJ2, respectively, and decreases to 62% and 60% where segment 2 starts. The covalent contributions are highly similar across the two systems. Only Arg422, in segments 1 and 2, and Met423, in the configurations close to the global minimum (end of segment 1 and beginning of segment 2: 4.5–6 Å) have strong covalent contributions. As Figure 8 shows, any strong V*_xc_* contributions to the ligand–pocket interaction are absent altogether after a distance of 11.7 Å. This means that *no V_xc_ contribution is responsible for the changes in the full PES from segment 2 onwards in either system.* Beyond segment 1, electrostatics becomes the main contributor of the interaction but only several residues have important contributions. Regarding the bromine atom, as shown in Figure 10, at a distance of 8.5–11 Å (segment 2 in 3CJ4 system and segment 3 in 3CJ2), the electrostatic contributions of Arg501 and His475 interact strongly for the first time with the bromine atom, at the same time that the interaction of bromine with Arg422 starts to decrease. The interaction of Arg501 and His475 reaches a maximum at a distance of 11 Å and then starts decreasing as well with distance. All the same interactions of bromine are also present in 3CJ4, where the full PES, shown in blue in Figure 4a, does not show a local maximum and minimum. The succinic acid chain of 3CJ4 has stronger electrostatic contributions and thus the bromine interactions, although present, are masked in the full PES. Nevertheless, the IQF calculation confirms the diagram shown in Figure 6. The strong interactions from Arg501 and His475 with the bromine atom of the ligands are not present in the closest configurations, where the bromine mainly interacts with Arg422 and Met265.

As the distance between the ligand and the pocket increases and approaches segment 3 (9 Å to 11 Å), it becomes clear that the interactions of the bromine atom with His475 and Arg501 become stronger. Meanwhile, the strong interaction with Arg422 (at the bottom of the site) becomes a plateau in the PES, precisely before reaching the distances of segment 3. It is important to remember that the concept of a segment was a result obtained by the REG-IQA method, not IQF, but the IQF results are supplemented by previous knowledge obtained using the REG-IQA method.

## 5. Discussion

This work marks an early example of an REG analysis of interactions between a protein and a ligand. The research question is “*Can IQA describe the interaction changes between similar drug candidates and the protein pocket*?” and our main research objective was to explore the limits of IQA and the REG method in a fragment-based drug design context, thereby addressing a problem of much bigger size than the realm of small molecules investigated predominantly with them. The changes in the interactions between the protein pocket and the inhibitors were assessed with the novel REG method. This method interprets, by computation, the energy profile of the total system as a function of the changing distance between the pocket and the drug candidates. REG pinpoints which atomic energy terms defined by IQA (sterics, exchange–correlation, or classical electrostatics) were more responsible for the total system behaviour during the separation of ligand and pocket. The most changing IQA terms, tracked as the highest REG value, highlight the pharmacophore in the ligand.

Relating our IQF numerical results to the experimental binding data of Antonysamy et al. [4], shown in Table 1, was not one of our objectives, because we had to remove solvent and most of the polymerase from our model. The contribution of the IQA partition to drug design, and to describing the affinities of a ligand binding to a target, is limited to the internal energy, which is part of the enthalpy. It is encouraging for future research into drug design based on IQA that a report considers the enthalpy optimisation as the main property to be optimised in drug design studies, as it is more difficult to optimise than the entropy component [51]. Work with isothermal titration calorimetry (ITC) data uses a relative comparison among systems within a narrow congeneric compound series [52]. Similarly, IQA is best used at this stage as a tool for comparing energy values between similar systems, such as the fragment-based drug design experiment that resulted in the HCV NS5B inhibitors 3CJ2 and 3CJ4. The differences in IQA are the best source of chemical insight when the structural changes between systems are known and follow progressive changes.

Being an early study of IQA in a fragment-based drug design setting, this study does not consider a solvent around the model or ligand. It does so only during the preparation stages, before the wave function is calculated via QM methods. The reason is that by removing the solvent, it is known for sure that the observed changes are caused only by the changes in a single variable: the distance between drug candidate and pocket. Solvent positions are not transferrable between systems, had they been considered, so they were removed altogether.

The experimentally calculated dissociation constants *K*_d_ for drug candidates 3CJ2 and 3CJ4 were 14 μM and 0.31 μM, respectively (i.e., a factor of 45 (=14/0.31) made 3CJ4 the better drug candidate). These values can be related to a Gibbs energy difference (ΔΔ*G* = −*RT* ln(45) = −(0.00831)(300)(3.81) = 9.5 kJmol^−1^ at 300 K). A value of about 10 kJ mol^−1^ is a small quantity in IQA terms but in terms of drug affinity is an important improvement. The problem is that a difference of less than 2 to 5 kJmol^−1^ between the IQA of a pair of equivalent atoms in different systems can arise for different reasons, such as the small variations in the environment, the level of theory or simply the integration procedure. A quantitative relationship cannot be constructed between the ligand affinities and the differences in IQA energies with the present calculations, because our calculations consider neither the full protein nor the solvent. A whole new set of calculations in a different level of theory could very well present variations close to 10 kJmol^−1^ because of the sum of small differences in the IQA calculation for each atom of the system.

Nevertheless, the REG method is a potent tool, capable of automatically ranking all atomic energies, both intra- and interatomic. REG generates two rankings: the ranking for the full atomic contributions (averaged *A-A′* ranking) with *n* REG values and the ranking of the pairwise contributions (*A-B* ranking) with *n^2^* REG values, where *n* is the number of atoms in the system. When considering only the pairwise *A-B* contributions ranking, the top contributions by themselves are not a good first approach to analyse the system because an atom can be involved in contributions both in favour of and against the system’s behaviour, both with comparably large REG values. The addition of all pairwise contributions gives the true contribution of a single atom, and considering only the strongest pairwise terms may be the equivalent of missing the wood for the trees. For these systems of considerable size, an REG value ranking of *A-A′ E_IQA_* (full atom contribution) already gives enough chemical insight about the system behaviour and the atoms that dominate it in the different segments. The pairwise *A-B* ranking can benefit from the *A-A′* ranking in order to avoid assuming that the atoms involved in the highest *A-B* REG values are automatically the ones with the highest atomic contribution. The *A-A′* ranking is a good first approach, while the *A-B* pairwise ranking is better suited for a more detailed analysis once the most representative atoms are known.

The REG-IQA method enabled the identification of a “*neighbour effect*” affecting the atoms involved in hydrogen bonds. The atoms covalently bonded to the hydrogen atom (the hydrogen donor) and to the hydrogen acceptor (the “neighbour of the hydrogen acceptor”) show an opposite trend in IQA changes to the hydrogen acceptor (the strongest contributor), and their energies become more negative when their neighbours are not involved in a hydrogen bond. Still, the favourable changes brought in by the hydrogen bond formation are larger, and keep the energies of the system more negative than when the hydrogen bond does not occur. This demonstrates that IQA shows how the system adapts to the changes brought in by the hydrogen bond formation and breakage. In these systems, where hydrogen bonds between the ligand and the protein pocket are numerous, the electrostatic (*V_cl_*) terms were the most important when the ligand and protein were far away. However, at short range (closer than the global minimum), the importance of *V_xc_* and *E_self_* increases.

This work also used the IQF method to analyse the interactions between the ligand and the pocket as fragments. The REG-IQA analysis and IQF have different strengths in the case study of the NS5B protein and two inhibitors. In REG-IQA, the method highlights the highest-contributing self-energies or pairwise interactions, depending on the PES segment. All is carried out automatically. The IQF analysis shows the sterics only in the deformation energy analysis, treating sterics and interaction energies as separate entities. Both REG-IQA and IQF use the IQA partition and need the same data to be applied but the obtained insight is different and complementary. REG-IQA has a fast and automatic way of highlighting the atoms whose sterics or interactions best represent the changes in a whole chemical system, but only IQF can describe interactions between ligands and pocket fragments, which is crucial in a drug design setting.

As a new arrival to computer-aided drug design and the well-established collection of methods analysing drug–protein interactions, it is natural to wonder what IQA brings to the table compared with traditional drug descriptors. This topological energy decomposition is robust and computationally expensive but its roots in electronic structure quantum calculations mean that it is naturally [53] atomistic, as opposed to energy decomposition methods based on perturbation theory, which can describe electronic effects such as bond breaking, bond formation, charge transfer and polarisation, while classifying the energies of the system according to different types of interaction. Several different energy decomposition analyses (EDA) have been formulated as a consequence of the lack of a unified definition of many chemical concepts (such as chemical bonding itself) and the alternative ways QM energies can be decomposed [54]. The methods called molecules-in-molecules (MIM3) [55] and ONETEP EDA [56] are examples of EDAs currently used in drug design, each using different approximations to work with thousands of atoms. It is important to note that the topological energy decomposition IQA used in this work does not use any shortcuts to include thousands of atoms but instead focuses on the ~120-atom system size in a full and proper QM analysis. A recent report increased the ligand–protein studied system size with IQA to the ~200 atoms mark [57].

The topological energy decomposition IQA proves to be powerful while describing non-covalent interactions. As a topological method that extracts insight directly from the wave function, IQA shows relevant information such as the type of chemical bonding, separating an interaction into exchange–correlation (covalency) and classical electrostatics, and the changes in the steric energy of an atom. In fact, recent developments allow IQA to be applied to a QM/MM case study [58], but even in such a method, the accuracy is increased by increasing the size of the QM region, among other things. The current trend in the IQA community is taking advantage of the increase in computer power and increasing the system sizes.

Cheminformatics techniques such as docking and quantitative structure–activity relationship (QSAR)/quantitative structure–property relationship (QSPR) are much less computationally expensive. Although there is the possibility of applying full flexibility in docking to the ligand and target interaction, its strongest point is arguably virtual screening, with just enough flexibility for the ligand and pocket to assess and rank the most promising ligands. It is important to note that docking methods must be validated for every new system.

On the other hand, QSAR and QSPR use different types of molecular descriptors (topological, electronic, ADMET, geometrical, and hybrid, to name a few), which are pre-calculated and obtained through a plethora of different methods, even machine learning [59], and through them, find correlations of activity and property with molecular structure, using the insight to optimise compounds for molecular design. The reliable prediction of these descriptors is significant for the development of predictable QSAR/QSPR models. QSAR/QSPR needs either careful descriptor selection by an experienced user, or the training of a model with machine learning. In contrast, IQA needs no reference values whatsoever, just a molecular conformation from which the wave function is calculated with a level of theory that the method can partition.

Finally, we mention the difference between IQA and molecular dynamics (MD) simulations. MD allows the calculation of progressive conformations through a period of time, giving the studied pocket and the ligand a conformational freedom with classical Newtonian mechanics. This approach enables the dynamic evolution of a molecular system, typically using a force field. The end result of an MD simulation is a series of conformations of a molecular system across a defined number of time steps. IQA takes the QM wave function calculated from a single conformation and essentially dissects all the interactions between each of its atoms. The topological EDA obtains energy values and contains everything that can be known about the studied system, as all behaviour and phenomena can be traced back to energy.

## 6. Conclusions

This work marks an early example of the relative energy gradient (REG) method being deployed in a drug design setting. The analysis shows that it can automatically highlight the pharmacophore, which consists of the ligand atoms that contribute the most to the binding. The method can also highlight the atoms of the pocket that contribute the most to binding, and which type of energy is most responsible for the interaction. Meanwhile, using the fragment-based topological energy decomposition IQF, the interaction between ligand and pocket is separated into exchange–correlation and electrostatic components, providing further details of this interaction and opening the door for the further optimisation of the drug candidates. The insight obtained from the REG-IQA and IQF analysis can be summarised as follows:

1. The changes in IQA energies along the progressive snapshots of a system where a coordinate of interest is changed (in this case, ligand–pocket distance) can explain and find the pharmacophore of a drug candidate from the interaction IQA data of a drug candidate (ligand) and protein pocket. The REG-IQA ranking determines which IQA terms can be used as a subset for explaining the full energetic behaviour of the system.
2. It was observed from the two types of REG ranking (*A-A′* and *A-B*) that the full atomic contributions ranking (*A-A′*) is a better first approach when looking at atomic contributions, as it only contains as many terms as the number of atoms in the system, i.e., it is a summary of the whole *A-B* ranking. In this ranking, an atom either has a contribution in favour of the whole system energetic profile or against it. The *A-B* interactions are more detailed but are not an appropriate first way of looking at a system this size or larger, as an atom can have contributions both in favour of and against the system’s energetic behaviour. We suggest *A-B* interaction energies to be considered only as a second step, for particularly important pairs of atoms whose energies are of interest and to find out which type of IQA energy is responsible for the importance of certain atoms.
3. Hydrogen bonds are very strong when compared with other electrostatic interactions. This work confirmed that new hydrogen bonds in improved drug candidates can mask other important interactions in the PES.
4. The addition of more electrostatic interactions, coming from the addition of a polar group in the drug design process, shifted the overall optimal position of the distance between the drug candidate and the pocket by 0.4 Å, as the global minimum will occur at the optimal distance considering all hydrogen bonds at the same time.
5. A *neighbour effect* was observed, in which an atom loses stability when it donates a proton or when its covalently bonded neighbour becomes a hydrogen acceptor. The energy rises when its covalent neighbour becomes involved in a hydrogen bond. This effect can only be observed in the full atomic contributions (*A-A′*) REG ranking, which summarises hundreds of IQA pairwise (*A-B*) terms. However, only by looking at the pairwise *A-B* ranking is it possible to observe that *V_cl_* is the type of IQA energy responsible for this effect.
6. The REG-IQA method reveals the importance of the self-energies in the system’s stabilisation when the ligand and the pocket approach each other too closely. A ranking of only the bromine *A-B* interactions reveals the importance of self-energy when a bromine atom is far enough and *E_self_* is more negative. This means that the heavy atom “would rather be by itself”.
7. The REG method allows the analysis of even a small increase in stability caused by the electrostatic attraction of positive residues interacting with the bromine atom of the ligand when it moves away from the allosteric site, and for us pinpoint that this behaviour comes mainly from electrostatics (*V_cl_*) and sterics (*E_self_*). Maybe the importance of electrostatics was to be expected considering the partial charge of the atom, but the importance of *E_self_* is a novel finding.
8. The IQF analysis obtains the ligand–pocket interaction energy in the IQA framework. At short range, the interaction has a predominantly covalent component until the separation of the global minimum, beyond which the contribution by *V_xc_* wanes and the electrostatics (*V_cl_*) take the predominant role in the waning interaction.
9. IQF confirms that the interaction between the ligand bromine atom and the pocket is largely similar across the two different systems, which supports the very concept of fragment-based drug design. The interactions of the moieties of earlier stages of a drug candidate are maintained as new moieties are added to improve them.
10. An IQA study allows for a thorough assessment of the enthalpic component in drug design, which is more difficult to optimise than the entropic contribution. A polar group in the drug candidate may help to bind it strongly to the pocket, but the surrounding solvent will also interact with this polar group and the entropic penalties coming from desolvation can decrease the binding. As drug candidates are currently being optimised both enthalpically and entropically, the importance of hydrophobic, poorly soluble drug candidates and a better description of the non-polar interactions makes IQA and QCT important newcomers to the field of drug design, which can contribute to the identification of more efficient drug candidates.

## Figures and Tables

**Table 1:** Structures and affinity data for the drug candidates discovered by Antonysamy et al. used in this work. Inspection of the structures shows that one (3CJ2) is the scaffold for the creation of the other (3CJ4).

PDB ID	Ligand Diagram	IC_50_ (μM)	K_d_ (μM)
3CJ2	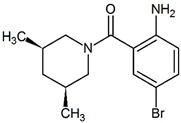	17	14
3CJ4	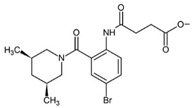	1	0.31

**Table 2:** Top (most positive) and bottom (most negative) full atomic contributions (*A-A′* ranking) by REG value for segment 1 in 3CJ4.

IQA Energy Term	Location	Partial Charge	REG Value	Pearson Correlation Coefficient
H91	His475 sidechain, interacting directly with the acid moiety of the ligand	Positive	0.164	0.997
S69	Met423, interacting directly with the ligand piperidine	Neutral	0.144	0.999
O47	Ligand acid moiety, interacting directly with His475 sidechain	Negative	0.100	0.998
H74	Met423, interacting directly with the ligand piperidine	Neutral	0.095	0.989
H30	Ligand piperidine, interacting directly with Met423	Neutral	0.083	0.995
C89	His475 sidechain, interacting directly with the acid moiety of the ligand	Neutral	0.075	0.975
H21	Ligand piperidine, interacting directly with Met423	Neutral	0.061	0.944
[…]	[…]	[…]	[…]	[…]
N93	His475 imidazole	Negative	−0.008	−0.638
O46	Ligand acid moiety	Negative	−0.008	−0.858
C61	Arg422 central carbon	Positive	−0.011	−0.958
C81	His475 main chain	Positive	−0.015	−0.912
H94	His475 imidazole	Positive	−0.018	−0.991

**Table 3:** The top pairwise interaction terms (*A-B* ranking) for segment 1 of 3CJ4, in favour of the system’s behaviour (top part, above the divider) and against the system’s behaviour (bottom part).

IQA Energy Term (A_B)	Location of A and of B, Separated by a Comma	Partial Charge	REG Value	Pearson Correlation Coefficient
*V_cl_*_Pair_C45_H91	Ligand (C45), His475 (H91)	Positive, neutral	0.327	0.995
*E_self_*_O47	Ligand	Negative	0.275	0.999
*E_self_*_S69	Met423	Neutral	0.225	0.996
*V_xc_*_Pair_C89_H91	His475, His475	Neutral, neutral	0.149	0.999
*E_self_*_H91	His475	Neutral	0.143	0.989
[…]	[…]		[…]	[…]
*V_xc_*_Pair_O47_H91	Ligand (O47), His475	Negative, neutral	−0.121	−0.990
*V_cl_*_Pair_O46_H91	Ligand (O46), His475	Negative, neutral	−0.152	−0.987
*V_cl_*_Pair_O47_H91	Ligand (O47), His475	Negative, neutral	−0.298	−0.996

**Table 4:** Top and bottom full atomic contributions (*A-A′* ranking) for segment 2 in 3CJ4.

IQA Energy Term	Location	Partial Charge	REG Value	Pearson Correlation Coefficient
O47	Ligand acid moiety (hydrogen acceptor)	Negative	0.800	0.997
O46	Ligand acid moiety (hydrogen acceptor)	Negative	0.498	0.998
O15	Ligand tertiary amide (hydrogen acceptor)	Negative	0.493	0.990
O38	Ligand secondary amide (hydrogen acceptor)	Negative	0.422	0.999
N8	Ligand secondary amide	Negative	0.335	0.990
N16	Ligand piperidine	Negative	0.237	0.961
[…]	[…]	[…]	[…]	[…]
N48	Arg501 (hydrogen donor)	Negative	−0.138	−0.957
N93	His475 imidazole (hydrogen donor)	Negative	−0.159	−0.886
O108	Ser476 hydroxyl (hydrogen donor)	Negative	−0.176	−0.863
N101	Ser476 main chain (hydrogen donor)	Negative	−0.220	−0.997
C14	Ligand tertiary amide (neighbour of hydrogen acceptor)	Positive	−0.375	−0.968
C10	Ligand secondary amide (neighbour of hydrogen acceptor)	Positive	−0.422	−0.994
C45	Ligand acid moiety (neighbour of hydrogen acceptor)	Positive	−0.555	−0.962

**Table 5:** The top pairwise interactions (*A-B* ranking) for segment 2 of 3CJ4, in favour of the system’s behaviour (top part) and against the system’s behaviour (bottom part). The highlighted rows represent the pairwise interaction between hydrogen acceptors and the hydrogen atom in hydrogen bonds.

IQA Energy Term (A_B)	Location of A and of B, Separated by a Comma	Partial Charge	REG Value	Pearson Correlation Coefficient
*V_cl_*_Pair_c45_n93	Ligand acid moiety (C45), His475 imidazole (N93)	Positive, negative	1.313	0.997
*V_cl_*_Pair_o47_h109	Ligand acid moiety (O47), Ser476 (H109)	Negative, positive	1.276	0.990
*V_cl_*_Pair_c45_o108	Ligand acid moiety(C45), Ser476 (O108)	Positive, negative	1.250	0.987
*V_cl_*_Pair_o47_h94	Ligand acid moiety (O47), His475 imidazole (H94)	Negative, positive	1.194	0.989
*V_cl_*_Pair_o15_c51	Ligand tertiary amide (O15), Arg501 (C51)	Negative, positive	1.151	0.981
*V_cl_*_Pair_c45_n101	Ligand acid moiety (C45), Ser476 main chain (N101)	Positive, negative	1.033	0.997
*V_cl_*_Pair_o47_c87	Ligand acid moiety (O47), His475 main chain (C87)	Negative, positive	0.962	0.992
*V_cl_*_Pair_c10_n101	Ligand secondary amide (C10), Ser476 main chain (N101)	Positive, negative	0.962	0.992
*V_cl_*_Pair_o38_c87	Ligand secondary amide (O38), His475 main chain (C87)	Negative, positive	0.868	0.995
*V_cl_*_Pair_c14_n48	Ligand tertiary amide (C14), Arg501 (N48)	Positive, negative	0.867	0.970
*V_cl_*_Pair_o47_c95	Ligand acid moiety (O47), His475 imidazole (C95)	Negative, positive	0.853	0.994
*V_cl_*_Pair_o15_h49	Ligand tertiary amide (O15), Arg501 (H49)	Negative, positive	0.846	0.988
[…]	[…]		[…]	[…]
*V_cl_*_Pair_c45_h94	Ligand acid moiety (C45), His475 imidazole (H94)	Positive, positive	1.013	−0.995
*V_cl_*_Pair_c45_h109	Ligand acid moiety (C45), Ser476 (H109)	Positive, positive	1.045	0.992
*V_cl_*_Pair_o38_n101	Ligand secondary amide (O38), Ser476 main chain (N101)	Negative, negative	1.057	−0.997
*V_cl_*_Pair_o47_n101	Ligand acid moiety (O47), Ser476 main chain (N101)	Negative, negative	1.071	−0.997
*V_cl_*_Pair_o15_n48	Ligand tertiary amide (O15), Arg501 (N48)	Negative, negative	1.150	−0.984
*V_cl_*_Pair_o47_o108	Ligand acid moiety (O47), Ser476 (O108)	Negative, negative	1.430	−0.992
*V_cl_*_Pair_o47_n93	Ligand acid moiety (O47), His475 imidazole (N93)	Negative, negative	1.449	−0.998

**Table 6:** Top and bottom REG values (*A-A′* rankings) for segments 2 and 4 in 3CJ2.

Segment 2				Segment 4			
IQA Energy Term	Location	Partial Charge	REG Value	IQA Energy Term	Location	Partial Charge	REG Value
O15	Ligand tertiary amide (hydrogen acceptor)	Negative	4.238	O15	Ligand tertiary amide (hydrogen acceptor)	Negative	4.399
C41	Arg501	Positive	2.326	N8	Ligand amino group (secondary amide in 3CJ4)	Negative	4.100
N8	Ligand amino group (secondary amide in 3CJ4)	Negative	1.811	H4	Ligand bromo-aryl	Neutral	3.943
H40	Arg501	Positive	1.513	N16	Ligand piperidine	Negative	2.981
N16	Ligand piperidine	Negative	1.257	C41	Arg501	Positive	0.860
H6	Ligand bromo-aryl	Neutral	1.046	C2	Ligand bromo-aryl	Positive	0.701
H44	Arg501	Positive	0.657	O98	Ser476 sidechain	Negative	0.408
H47	Arg501	Positive	0.643	C51	Arg422	Positive	0.355
H46	Arg501	Positive	0.580	C71	Leu474	Positive	0.350
[…]	[…]	[…]	[…]	[…]	[…]	[…]	[…]
C11	Ligand bromo-aryl	Neutral	−0.682	N38	Arg501 (hydrogen donor)	Negative	−0.915
N38	Arg501 (hydrogen donor)	Negative	−0.732	C35	Ligand dimethyl piperidine	Positive	−0.946
H12	Ligand bromo-aryl	Neutral	−0.793	C17	Ligand dimethyl piperidine	Positive	−1.004
N45	Arg501	Negative	−0.988	H9	Ligand primary amine	Positive	−1.688
N42	Arg501	Negative	−1.030	H10	Ligand primary amine	Positive	−1.779
H39	Arg501	Positive	−1.307	C7	Ligand primary amine	Positive	−1.795
H4	Ligand bromo-aryl	Neutral	−1.607	Br1	Ligand bromo-aryl	Negative	−2.539
C14	Ligand tertiary amide (neighbour of hydrogen acceptor)	Positive	−2.263	C14	Ligand tertiary amide (neighbour of hydrogen acceptor)	Positive	−4.595

**Table 7:** Top and bottom REG values (*A-A′* ranking) for segment 3 in 3CJ2. The bromine contribution is highlighted.

IQA Energy Term	Location	Partial Charge	REG Value	Pearson Correlation Coefficient
C14	Ligand, tertiary amide (neighbour to Hydrogen acceptor)	Positive	4.404	0.948
Br1	Ligand, bromo-aryl moiety	Negative	4.335	0.954
N38	Arg501 (hydrogen donor)	Negative	1.701	0.978
H10	Ligand, primary amine	Positive	1.571	0.940
C7	Ligand, bromo-aryl moiety (bonded to amino group)	Positive	1.502	0.935
H9	Ligand, primary amine	Positive	1.398	0.936
H4	Ligand, bromo-aryl moiety	Positive	1.136	0.723
C17	Ligand, dimethylpiperidine	-	0.931	0.946
C35	Ligand, dimethylpiperidine	-	0.909	0.948
[…]	[…]	[…]	[…]	[…]
C41	Arg501	Positive	−1.529	−0.983
H6	Ligand, bromo-aryl moiety	-	−2.502	−0.997
N16	Ligand, dimethylpiperidine	Negative	−2.822	−0.947
N8	Ligand, amino group bonded to bromo-aryl moiety	Negative	−4.048	−0.951
O15	Ligand, tertiary amide (hydrogen acceptor)	Negative	−5.477	−0.964

**Table 8:** The top pairwise interaction terms involving the bromine atom (*A-B* ranking), for segment 3 in 3CJ2, in favour of the system’s behaviour (top part) and against the system’s behaviour (bottom part).

IQA Energy Term (A_B)	Location of A and of B, Separated by a Comma	Partial Charge	REG Value	Pearson Correlation Coefficient
*V_cl_*_Pair_br1_c77	Ligand, His475	Negative, positive	2.995	0.959
*V_cl_*_Pair_br1_c41	Ligand, Arg501	Negative, positive	2.924	0.960
*V_cl_*_Pair_br1_c100	Ligand, Ser476	Negative, positive	2.299	0.942
*E_self_*_br1	Ligand	Negative	2.247	0.952
*V_cl_*_Pair_br1_c14	Ligand (both)	Negative, positive	1.739	0.947
*V_cl_*_Pair_br1_h92	Ligand, Ser476	Negative, positive	1.655	0.930
*V_cl_*_Pair_br1_c85	Ligand, His475	Negative, positive	1.587	0.935
[…]	[…]		[…]	[…]
*V_cl_*_Pair_br1_o78	Ligand, His475	Negative, negative	−1.730	−0.956
*V_cl_*_Pair_br1_o98	Ligand, Ser476	Negative, negative	−1.841	−0.909
*V_cl_*_Pair_br1_n102	Ligand, Ser476	Negative, negative	−1.927	−0.948
*V_cl_*_Pair_br1_n83	Ligand, His475	Negative, negative	−2.304	−0.933
*V_cl_*_Pair_br1_n38	Ligand, Arg501	Negative, negative	−2.863	−0.961
*V_cl_*_Pair_br1_n91	Ligand, Ser476	Negative, negative	−3.438	−0.949

## Data Availability

Data is contained within the article and Supplementary Materials.

## Supplementary Materials

The following supporting information can be downloaded at: https://www.mdpi.com/article/10.3390/ph15101237/s1. Figure S1: Graphical representation of the progressively bigger pocket surrounding the 3CJ4 ligand. Figure S2: Progression of E_self_ with increasing system size for the 3CJ4 ligand atoms. Figure S3: Progression of V_xc_ with increasing system size for the 3CJ4 ligand atoms. Figure S4: Progression of V_cl_ with increasing system size for the 3CJ4 ligand atoms. Figure S5: Diagram depicting the reported distances from the ligand to the bottom of the pocket. Figure S6: Diagram highlighting the atoms that clash the most between the ligand and the pocket in the closest configuration of 3CJ4. Figure S7: The deformation energies for the 3CJ4 and the 3CJ2 across the studied configurations. Figure S8: Improvement of 3CJ4 ligand–pocket E_inter_ compared with 3CJ2 ligand–pocket E_inter_. Table S1: The residues considered in each iteration of the size of the allosteric site model assessment. Table S2: Distance notation used in this work for the 17 configurations of 3CJ4. Table S3: Distance notation used in this work for the 22 configurations of 3CJ2. Table S4: The top atoms (*A-A′* ranking) by absolute REG values for segment 1 of 3CJ2. Table S5: The top pairwise interactions (*A-B* ranking) for segment 1 of 3CJ2, in favour of and against the system energetic behaviour. Table S6: The top pairwise interactions (*A-B* ranking) for segment 2 of 3CJ2, in favour of and against the system energetic behaviour. Table S7: The top pairwise interactions (*A-B* ranking) for segment 4 of 3CJ2, in favour of and against the system energetic behaviour. Table S8: The top pairwise interactions (*A-B* ranking) for segment 3 of 3CJ2, in favour of and against the system’s energetic behaviour.
