# Supplementary material for: The IQA Energy Partition in a Drug Design Setting: A Hepatitis C Virus RNA-Dependent RNA Polymerase (NS5B) Case Study
Source: Pharmaceuticals (Basel). 2022 Oct 8;15(10):1237. doi: 10.3390/ph15101237 (PMC9609620; doi:10.3390/ph15101237)
Supplement: Supplementary file 1 [file pharmaceuticals-15-01237-s001.zip › pharmaceuticals-1927691-supplementary.pdf]

# The IQA Energy Partition in a Drug Design Setting: A Hepatitis C Virus RNA-Dependent RNA Polymerase (NS5B) Case Study

**César A. Zapata-Acevedo [1,2,3] and Paul L. A. Popelier [1,*]**

[1] Department of Chemistry, University of Manchester, Oxford Road, Manchester M13 9PL, UK
[2] Instituto de Química, Universidad Nacional Autónoma de México, Circuito Exterior s/n, Ciudad Universitaria, Coyoacán, Ciudad de México 04510, Mexico
[3] Tecnológico de Monterrey, Campus Santa Fe, Av. Carlos Lazo 100, Santa Fe, La Loma, Álvaro Obregón, Ciudad de México 01389, Mexico
* Correspondence: pla@manchester.ac.uk; Tel.: +44-161-3064-511

# Contents

**1. Selection of the pocket model size** .......... 3

**2. Examples of the configurations of the studied systems in PDB format** .......... 7

2.1 PDB file of a frame of the 3CJ4 system progression. .......... 7

2.2 PDB file of a frame of the 3CJ2 system progression .......... 10

**3. Explanation of the reported distance notation** .......... 13

**4. REG ranking graphs** .......... 15

4.1 The 3CJ4 System .......... 15

4.2 The 3CJ2 system .......... **Error! Bookmark not defined.**

**5. Interacting Quantum Fragments Ligand-Pocket Analysis** .......... 21

# 1. Selection of the pocket model size

The determination of the number of atoms that would represent the NS5B protein allosteric thumb site II was performed by calculating the IQA energies for the ligand atoms only, in an increasingly larger representation of the active site. A calculation by the program AIMAll does not have an imposed size limit but works with the computational power available. With our hardware at the time, our computational experiments show a size limit at a 400-atom system (a number that must contain both the ligand and the protein pocket model).

The creation of the iteratively smaller systems started from the minimised, protonated structure of the Protein Data Bank's 3CJ4 structure. A massive 369-atom system was obtained, in which we maintained every residue that contained at least one atom in a 4.5 Å distance from any ligand atom.

The atoms in the system were fixed, which means that the position of the atoms were in all cases an accurate representation of their positions in the crystal, and every smaller system was cut from the previous one. Special care was taken to remove, at the last stage, the residues that affected the charge of the system. Figure S1 shows a 2D representation of the residues surrounding the drug candidate. Table S1 provides more details of the residues labelled in Figure 1.

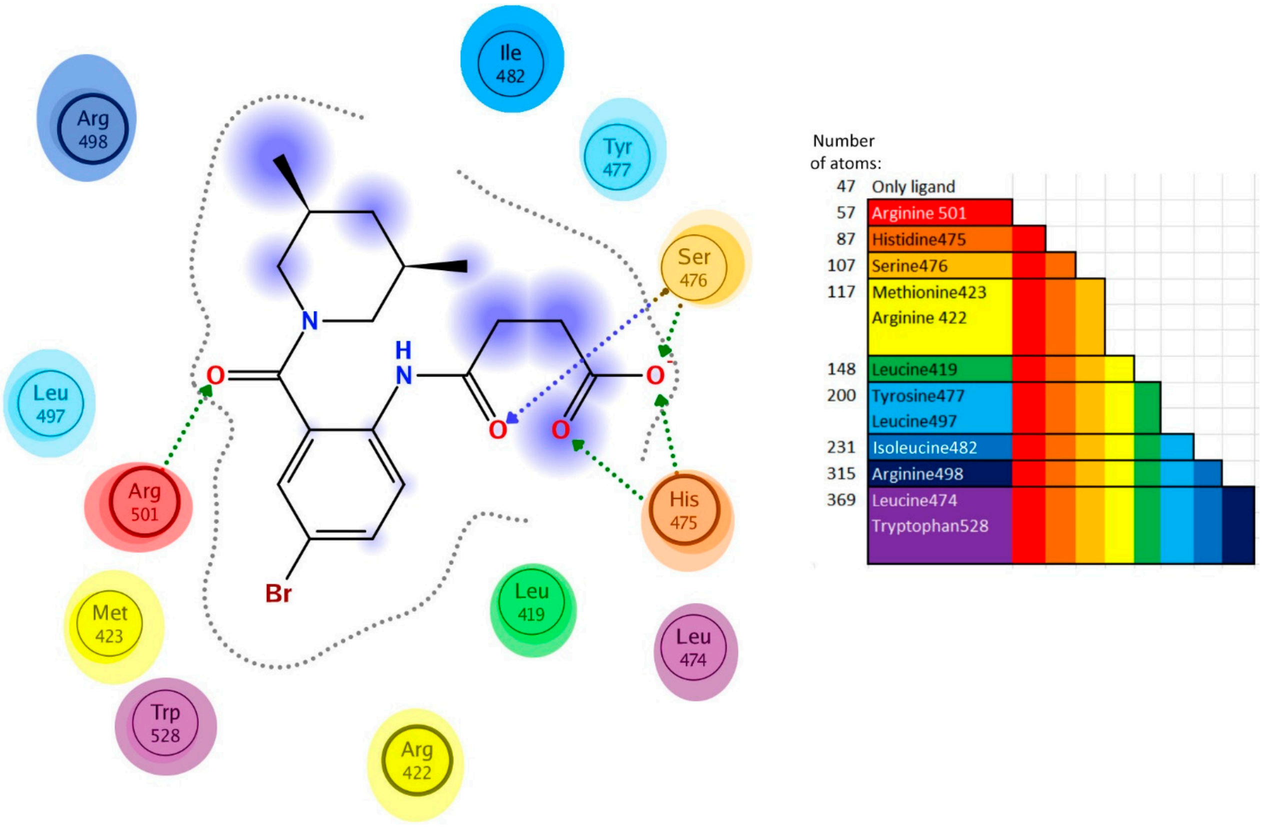


**Figure S1.** Graphical representation of the progressively bigger pocket surrounding the 3CJ4 ligand. Starting with red, every new rainbow colour includes at least one new residue, the ligand and all of the previous residues until reaching the 10$^{th}$ system, which has 369 atoms in total.

| Total number of atoms | Atoms in ligand | Atoms in pocket | Residues included in the model | | | | | | | | | | | |
|---|---|---|---|---|---|---|---|---|---|---|---|---|---|---|
| 47 | 47 | 0 | None | | | | | | | | | | | |
| 57 | 47 | 10 | Arg 501 | | | | | | | | | | | |
| 87 | 47 | 40 | Arg 501 | His 475 | | | | | | | | | | |
| 107 | 47 | 60 | Arg 501 | His 475 | Ser 476 | | | | | | | | | |
| 117 | 47 | 70 | Arg 501 | His 475 | Ser 476 | Met 423 | Arg 422 | | | | | | | |
| 148 | 47 | 101 | Arg 501 | His 475 | Ser 476 | Met 423 | Arg 422 | Leu 419 | | | | | | |
| 200 | 47 | 153 | Arg 501 | His 475 | Ser 476 | Met 423 | Arg 422 | Leu 419 | Tyr 477 | Leu 497 | | | | |
| 231 | 47 | 184 | Arg 501 | His 475 | Ser 476 | Met 423 | Arg 422 | Leu 419 | Tyr 477 | Leu 497 | Ile 482 | | | |
| 315 | 47 | 268 | Arg 501 | His 475 | Ser 476 | Met 423 | Arg 422 | Leu 419 | Tyr 477 | Leu 497 | Ile 482 | Arg 498 | | |
| 369 | 47 | 322 | Arg 501 | His 475 | Ser 476 | Met 423 | Arg 422 | Leu 419 | Tyr 477 | Leu 497 | Ile 482 | Arg 498 | Leu 474 | Trp 528 |

**Table S1**. The residues considered in each iteration of the size of the allosteric site model assessment. Highlighted in blue is the 70-atom environment, which, along with the 47 atoms of the ligand, forms the system of 117 atoms that is used in the computational experiments in the main text.

The number of atoms in the pocket model increases in certain discrete numbers. Moreover, some residues can be accounted for only with the side chain, if the backbone is far away. Arginines can be accounted for with guanidinium ions. The atomic integrations of the bromine atom had difficulty being completed in the larger systems. Since the atomic integrations for bromine in the largest systems (315 and 369 atoms, respectively) took more than 2 weeks, those calculations were omitted. After having carried out the IQA calculations for all 10 systems, the 117 atoms system was selected as the smallest reliable model: a plateau is reached at the 70-atoms environment (the size of the 3CJ4 ligand is 47 atoms and the size of the 3CJ2 ligand is 37). The IQA energies could change quantitatively with increasing system size but, importantly, considering residues further out did not change the ranking of IQA values. Figures S2, S3 and S4 show that the plateau is reached for all three types of IQA energy, each figure focusing on one energy type.

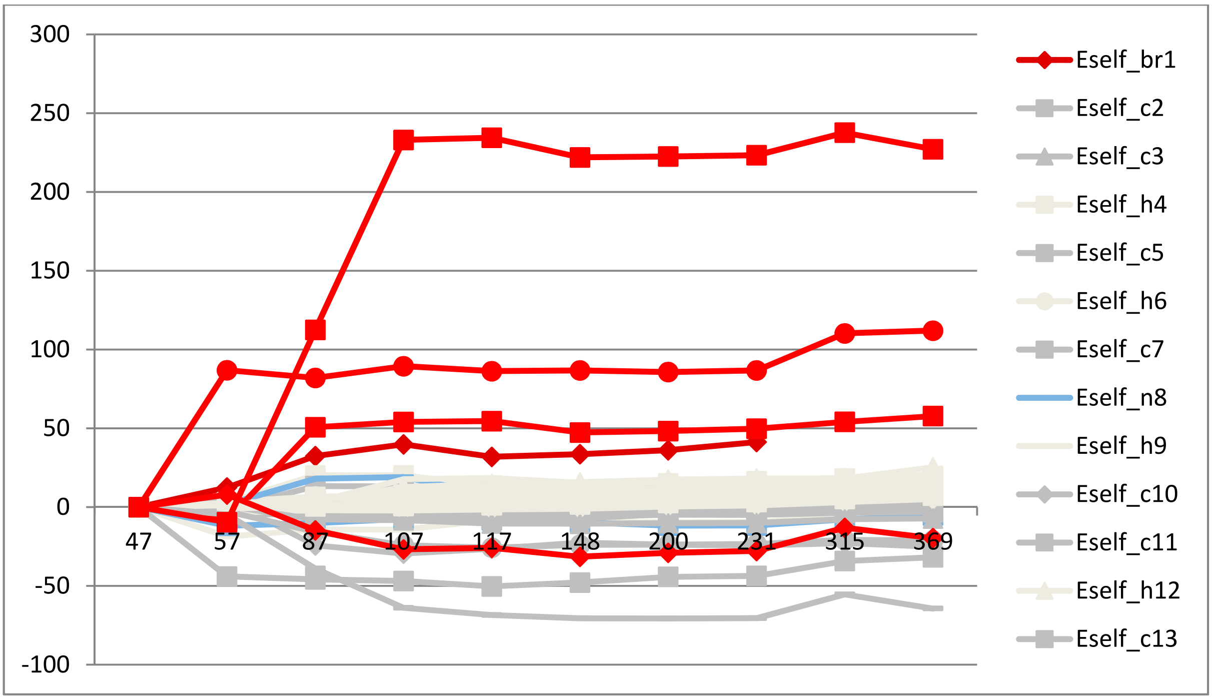


**Figure S2.** Progression of $E_{self}$ with increasing system size for the 3CJ4 ligand atoms.

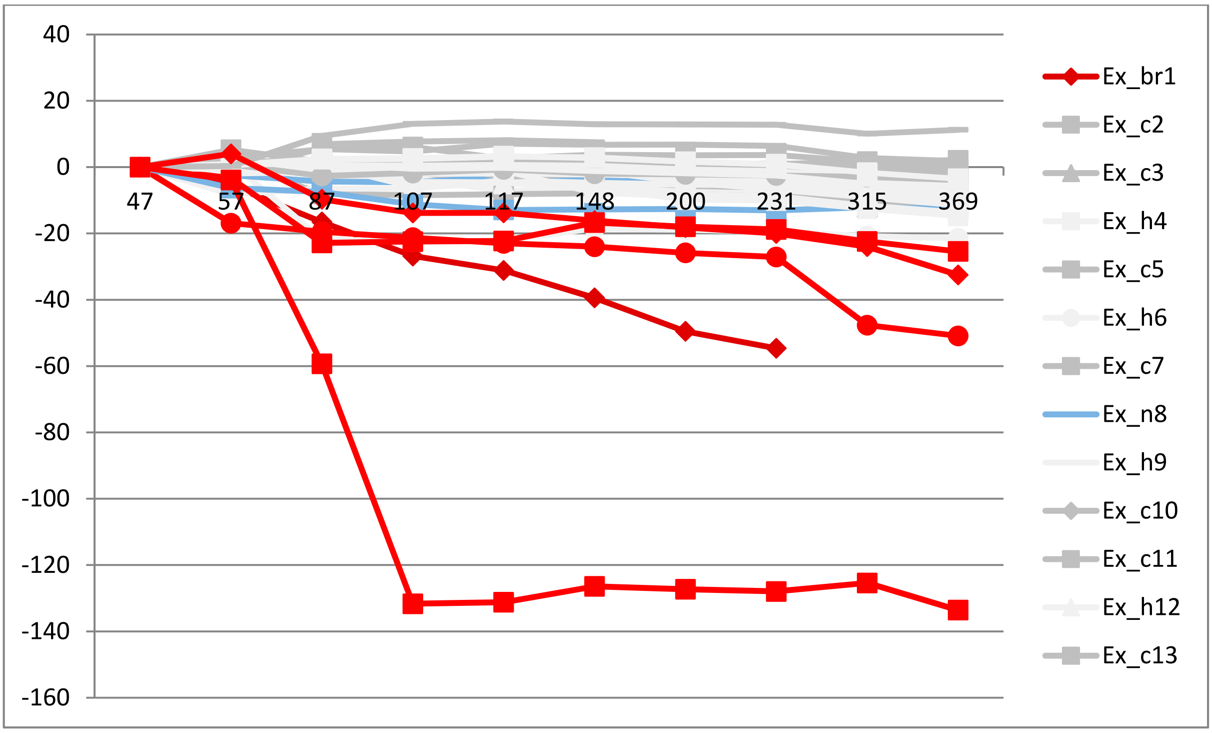


**Figure S3.** Progression of $V_{xc}$ with increasing system size for the 3CJ4 ligand atoms.

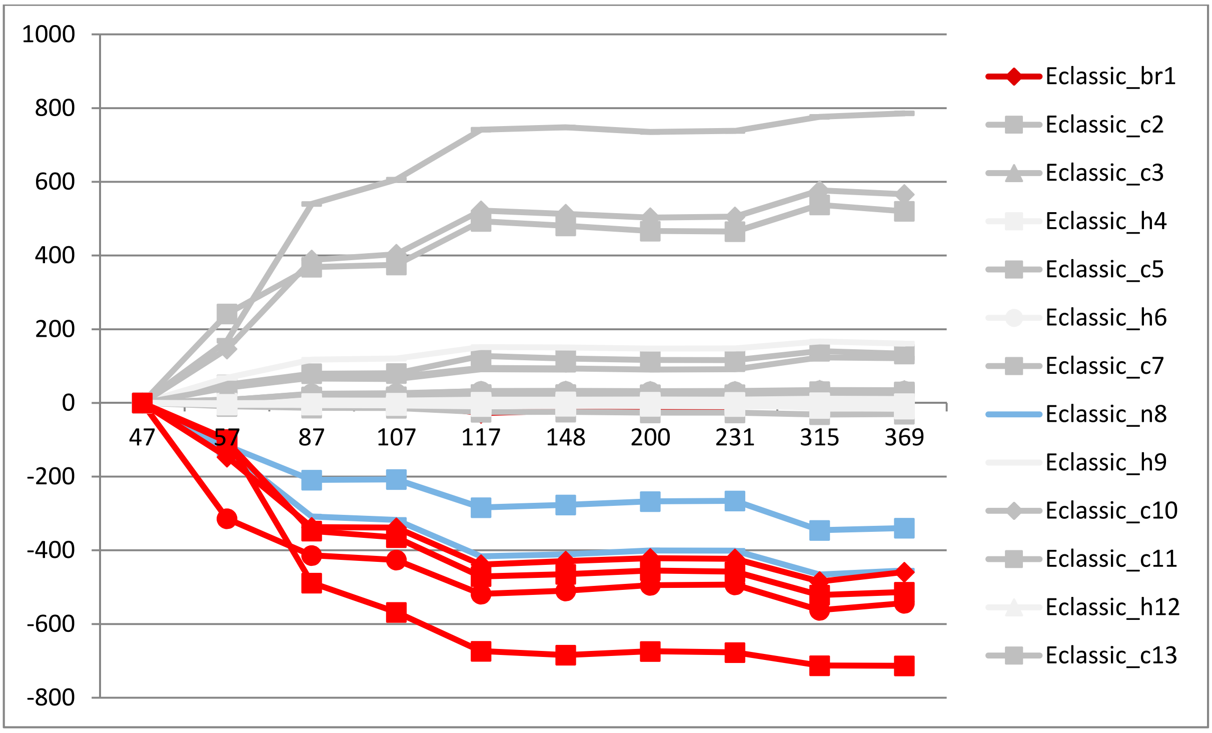


**Figure S4.** Progression of $V_{cl}$ with increasing system size for the 3CJ4 ligand atoms.

## 2. Examples of the configurations of the studied systems in PDB format

### 2.1 PDB file of a frame of the 3CJ4 system progression.

```
TITLE     NEWFULL_FROM_NEWHUGE_B3LYP_11_3_fixed
REMARK   1 File created by GaussView 5.0.9
HETATM    1 BR1  SX5 A 571     -19.939   8.741 -12.912                       Br
HETATM    2  C11 SX5 A 571     -21.654   8.053 -13.286                        C
HETATM    3  C12 SX5 A 571     -21.912   6.689 -13.140                        C
HETATM    4  H12 SX5 A 571     -21.134   6.037 -12.832                        H
HETATM    5  C13 SX5 A 571     -23.184   6.193 -13.395                        C
HETATM    6  H13 SX5 A 571     -23.400   5.170 -13.278                        H
HETATM    7  C14 SX5 A 571     -24.193   7.053 -13.822                        C
HETATM    8  N2  SX5 A 571     -25.455   6.519 -14.157                        N
HETATM    9  H2  SX5 A 571     -25.837   6.769 -15.093                        H
HETATM   10  C15 SX5 A 571     -26.228   5.669 -13.406                        C
HETATM   11  C10 SX5 A 571     -22.665   8.913 -13.699                        C
HETATM   12  H10 SX5 A 571     -22.459   9.945 -13.808                        H
HETATM   13  C9  SX5 A 571     -23.950   8.432 -13.974                        C
HETATM   14  C8  SX5 A 571     -24.994   9.351 -14.412                        C
HETATM   15  O1  SX5 A 571     -24.990   9.813 -15.557                        O
HETATM   16  N1  SX5 A 571     -26.022   9.720 -13.542                        N
HETATM   17  C6  SX5 A 571     -26.188   9.042 -12.219                        C
HETATM   18  H61 SX5 A 571     -26.944   8.273 -12.306                        H
HETATM   19  H62 SX5 A 571     -25.249   8.643 -11.859                        H
HETATM   20  C4  SX5 A 571     -26.654  10.096 -11.189                        C
HETATM   21  H4  SX5 A 571     -25.896  10.865 -11.042                        H
HETATM   22  C5  SX5 A 571     -26.912   9.385  -9.850                        C
HETATM   23  H52 SX5 A 571     -27.247  10.103  -9.102                        H
HETATM   24  H51 SX5 A 571     -27.678   8.616  -9.970                        H
HETATM   25  H53 SX5 A 571     -26.000   8.903  -9.497                        H
HETATM   26  C3  SX5 A 571     -27.948  10.752 -11.689                        C
HETATM   27  H32 SX5 A 571     -28.723   9.994 -11.820                        H
HETATM   28  H31 SX5 A 571     -28.289  11.483 -10.954                        H
HETATM   29  C2  SX5 A 571     -27.672  11.457 -13.025                        C
HETATM   30  H2  SX5 A 571     -26.913  12.224 -12.876                        H
HETATM   31  C1  SX5 A 571     -28.958  12.122 -13.530                        C
HETATM   32  H12 SX5 A 571     -28.766  12.617 -14.482                        H
HETATM   33  H11 SX5 A 571     -29.746  11.378 -13.665                        H
HETATM   34  H13 SX5 A 571     -29.292  12.866 -12.807                        H
HETATM   35  C7  SX5 A 571     -27.220  10.416 -14.074                        C
HETATM   36  H72 SX5 A 571     -28.008   9.690 -14.226                        H
HETATM   37  H71 SX5 A 571     -26.980  10.951 -14.982                        H
HETATM   38  O2  SX5 A 571     -25.798   5.175 -12.378                        O
HETATM   39  C16 SX5 A 571     -27.633   5.428 -13.936                        C
HETATM   40 H162 SX5 A 571     -27.792   6.023 -14.824                        H
HETATM   41 H161 SX5 A 571     -28.339   5.677 -13.155                        H
HETATM   42  C17 SX5 A 571     -27.809   3.934 -14.307                        C
HETATM   43 H171 SX5 A 571     -28.652   3.810 -14.985                        H
HETATM   44 H172 SX5 A 571     -27.996   3.360 -13.400                        H
HETATM   45  C18 SX5 A 571     -26.544   3.411 -14.974                        C
HETATM   46  O3  SX5 A 571     -26.083   4.036 -15.961                        O
```

```
HETATM   47  O4  SX5 A 571     -26.011   2.415 -14.422                   O
ATOM     48  NH1 ARG A 501     -24.651   9.015 -18.146                   N
ATOM     49 HH12 ARG A 501     -24.806   9.472 -17.241                   H
ATOM     50 HH11 ARG A 501     -24.870   8.033 -18.211                   H
ATOM     51  CZ  ARG A 501     -24.106   9.698 -19.134                   C
ATOM     52  NE  ARG A 501     -23.819  10.988 -19.008                   N
ATOM     53  H   ARG A 501     -24.013  11.580 -18.206                   H
ATOM     54  HE  ARG A 501     -23.301  11.391 -19.783                   H
ATOM     55  NH2 ARG A 501     -23.820   9.117 -20.277                   N
ATOM     56 HH22 ARG A 501     -23.934   8.126 -20.376                   H
ATOM     57 HH21 ARG A 501     -23.340   9.649 -21.003                   H
ATOM     58  NE  ARG A 422     -16.213   7.525 -12.964                   N
ATOM     59  HE  ARG A 422     -15.656   8.121 -13.572                   H
ATOM     60  H   ARG A 422     -16.148   7.677 -11.960                   H
ATOM     61  CZ  ARG A 422     -16.944   6.619 -13.596                   C
ATOM     62  NH1 ARG A 422     -17.721   5.782 -12.949                   N
ATOM     63 HH12 ARG A 422     -17.789   5.857 -11.948                   H
ATOM     64 HH11 ARG A 422     -18.309   5.116 -13.438                   H
ATOM     65  NH2 ARG A 422     -16.907   6.578 -14.905                   N
ATOM     66 HH21 ARG A 422     -16.224   7.188 -15.364                   H
ATOM     67 HH22 ARG A 422     -17.568   5.994 -15.429                   H
ATOM     68  CG  MET A 423     -21.355  12.148 -11.777                   C
ATOM     69  SD  MET A 423     -23.154  11.970 -11.700                   S
ATOM     70  CE  MET A 423     -23.633  12.795 -13.233                   C
ATOM     71  HG2 MET A 423     -20.900  11.373 -11.162                   H
ATOM     72  HG3 MET A 423     -21.038  11.989 -12.807                   H
ATOM     73  H   MET A 423     -21.001  13.117 -11.430                   H
ATOM     74  HE1 MET A 423     -24.716  12.893 -13.265                   H
ATOM     75  HE2 MET A 423     -23.313  12.193 -14.079                   H
ATOM     76  HE3 MET A 423     -23.177  13.784 -13.286                   H
ATOM     77  CA  LEU A 474     -18.070   2.266 -12.702                   C
ATOM     78  H   LEU A 474     -17.333   2.432 -13.485                   H
ATOM     79  H   LEU A 474     -17.732   2.579 -11.715                   H
ATOM     80  HA  LEU A 474     -18.300   1.200 -12.671                   H
ATOM     81  C   LEU A 474     -19.375   3.014 -13.025                   C
ATOM     82  O   LEU A 474     -19.357   4.106 -13.616                   O
ATOM     83  N   HIS A 475     -20.504   2.464 -12.571                   N
ATOM     84  H   HIS A 475     -20.456   1.556 -12.103                   H
ATOM     85  CA  HIS A 475     -21.827   3.074 -12.708                   C
ATOM     86  HA  HIS A 475     -21.730   4.129 -12.467                   H
ATOM     87  C   HIS A 475     -22.814   2.469 -11.699                   C
ATOM     88  O   HIS A 475     -22.443   1.557 -10.958                   O
ATOM     89  CB  HIS A 475     -22.305   2.907 -14.169                   C
ATOM     90  HB2 HIS A 475     -21.554   3.310 -14.848                   H
ATOM     91  HB3 HIS A 475     -23.212   3.490 -14.324                   H
ATOM     92  CG  HIS A 475     -22.606   1.485 -14.561                   C
ATOM     93  ND1 HIS A 475     -23.870   0.970 -14.748                   N
ATOM     94  HD1 HIS A 475     -24.736   1.531 -14.679                   H
ATOM     95  CE1 HIS A 475     -23.768  -0.352 -14.961                   C
ATOM     96  HE1 HIS A 475     -24.596  -1.043 -15.071                   H
ATOM     97  NE2 HIS A 475     -22.462  -0.675 -14.949                   N
ATOM     98  HE2 HIS A 475     -22.071  -1.623 -14.992                   H
ATOM     99  CD2 HIS A 475     -21.715   0.460 -14.705                   C
```

```
ATOM    100  HD2 HIS A 475     -20.639   0.517 -14.563                    H
ATOM    101  N   SER A 476     -24.063   2.954 -11.674                    N
ATOM    102  H   SER A 476     -24.324   3.716 -12.289                    H
ATOM    103  CA  SER A 476     -25.123   2.403 -10.826                    C
ATOM    104  HA  SER A 476     -25.944   3.115 -10.828                    H
ATOM    105  CB  SER A 476     -25.671   1.110 -11.447                    C
ATOM    106  HB3 SER A 476     -24.857   0.506 -11.846                    H
ATOM    107  HB2 SER A 476     -26.187   0.532 -10.679                    H
ATOM    108  OG  SER A 476     -26.611   1.392 -12.466                    O
ATOM    109  H   SER A 476     -26.148   1.616 -13.276                    H
ATOM    110  C   SER A 476     -24.674   2.243  -9.360                    C
ATOM    111  O   SER A 476     -24.525   1.122  -8.863                    O
ATOM    112  N   TYR A 477     -24.443   3.376  -8.684                    N
ATOM    113  H   TYR A 477     -24.627   4.254  -9.166                    H
ATOM    114  CA  TYR A 477     -24.157   3.486  -7.250                    C
ATOM    115  HA  TYR A 477     -23.432   2.720  -6.976                    H
ATOM    116  H   TYR A 477     -23.712   4.464  -7.074                    H
ATOM    117  H   TYR A 477     -25.055   3.323  -6.655                    H
END
```

2.2 PDB file of a frame of the 3CJ2 system progression

```
TITLE     3CJ2F3CJ4_B3LYP_11_3_fixed
REMARK   1 File created by GaussView 5.0.9
HETATM    1 BR1  SX5 A 571     -19.939   8.741 -12.912                      Br
HETATM    2  C11 SX5 A 571     -21.654   8.053 -13.286                       C
HETATM    3  C12 SX5 A 571     -21.912   6.689 -13.140                       C
HETATM    4  H12 SX5 A 571     -21.134   6.037 -12.832                       H
HETATM    5  C13 SX5 A 571     -23.184   6.193 -13.395                       C
HETATM    6  H13 SX5 A 571     -23.400   5.170 -13.278                       H
HETATM    7  C14 SX5 A 571     -24.193   7.053 -13.822                       C
HETATM    8  N2  SX5 A 571     -25.455   6.519 -14.157                       N
HETATM    9  H2  SX5 A 571     -25.899   6.727 -15.075                       H
HETATM   10 C15  SX5 A 571     -25.956   5.879 -13.505                       H
HETATM   11  C10 SX5 A 571     -22.665   8.913 -13.699                       C
HETATM   12  H10 SX5 A 571     -22.459   9.945 -13.808                       H
HETATM   13  C9  SX5 A 571     -23.950   8.432 -13.974                       C
HETATM   14  C8  SX5 A 571     -24.994   9.351 -14.412                       C
HETATM   15  O1  SX5 A 571     -24.990   9.813 -15.557                       O
HETATM   16  N1  SX5 A 571     -26.022   9.720 -13.542                       N
HETATM   17  C6  SX5 A 571     -26.188   9.042 -12.219                       C
HETATM   18  H61 SX5 A 571     -26.944   8.273 -12.306                       H
HETATM   19  H62 SX5 A 571     -25.249   8.643 -11.859                       H
HETATM   20  C4  SX5 A 571     -26.654  10.096 -11.189                       C
HETATM   21  H4  SX5 A 571     -25.896  10.865 -11.042                       H
HETATM   22  C5  SX5 A 571     -26.912   9.385  -9.850                       C
HETATM   23  H52 SX5 A 571     -27.247  10.103  -9.102                       H
HETATM   24  H51 SX5 A 571     -27.678   8.616  -9.970                       H
HETATM   25  H53 SX5 A 571     -26.000   8.903  -9.497                       H
HETATM   26  C3  SX5 A 571     -27.948  10.752 -11.689                       C
HETATM   27  H32 SX5 A 571     -28.723   9.994 -11.820                       H
HETATM   28  H31 SX5 A 571     -28.289  11.483 -10.954                       H
HETATM   29  C2  SX5 A 571     -27.672  11.457 -13.025                       C
HETATM   30  H2  SX5 A 571     -26.913  12.224 -12.876                       H
HETATM   31  C1  SX5 A 571     -28.958  12.122 -13.530                       C
HETATM   32  H12 SX5 A 571     -28.766  12.617 -14.482                       H
HETATM   33  H11 SX5 A 571     -29.746  11.378 -13.665                       H
HETATM   34  H13 SX5 A 571     -29.292  12.866 -12.807                       H
HETATM   35  C7  SX5 A 571     -27.220  10.416 -14.074                       C
HETATM   36  H72 SX5 A 571     -28.008   9.690 -14.226                       H
HETATM   37  H71 SX5 A 571     -26.980  10.951 -14.982                       H
ATOM     38  NH1 ARG A 501     -24.651   9.015 -18.146                       N
ATOM     39 HH12 ARG A 501     -24.806   9.472 -17.241                       H
ATOM     40 HH11 ARG A 501     -24.870   8.033 -18.211                       H
ATOM     41  CZ  ARG A 501     -24.106   9.698 -19.134                       C
ATOM     42  NE  ARG A 501     -23.819  10.988 -19.008                       N
ATOM     43  H   ARG A 501     -24.013  11.580 -18.206                       H
ATOM     44  HE  ARG A 501     -23.301  11.391 -19.783                       H
ATOM     45  NH2 ARG A 501     -23.820   9.117 -20.277                       N
ATOM     46 HH22 ARG A 501     -23.934   8.126 -20.376                       H
ATOM     47 HH21 ARG A 501     -23.340   9.649 -21.003                       H
ATOM     48  NE  ARG A 422     -16.213   7.525 -12.964                       N
ATOM     49  HE  ARG A 422     -15.656   8.121 -13.572                       H
```

```
ATOM     50  H   ARG A 422     -16.148   7.677 -11.960                       H
ATOM     51  CZ  ARG A 422     -16.944   6.619 -13.596                       C
ATOM     52  NH1 ARG A 422     -17.721   5.782 -12.949                       N
ATOM     53 HH12 ARG A 422     -17.789   5.857 -11.948                       H
ATOM     54 HH11 ARG A 422     -18.309   5.116 -13.438                       H
ATOM     55  NH2 ARG A 422     -16.907   6.578 -14.905                       N
ATOM     56 HH21 ARG A 422     -16.224   7.188 -15.364                       H
ATOM     57 HH22 ARG A 422     -17.568   5.994 -15.429                       H
ATOM     58  CG  MET A 423     -21.355  12.148 -11.777                       C
ATOM     59  SD  MET A 423     -23.154  11.970 -11.700                       S
ATOM     60  CE  MET A 423     -23.633  12.795 -13.233                       C
ATOM     61  HG2 MET A 423     -20.900  11.373 -11.162                       H
ATOM     62  HG3 MET A 423     -21.038  11.989 -12.807                       H
ATOM     63  H   MET A 423     -21.001  13.117 -11.430                       H
ATOM     64  HE1 MET A 423     -24.716  12.893 -13.265                       H
ATOM     65  HE2 MET A 423     -23.313  12.193 -14.079                       H
ATOM     66  HE3 MET A 423     -23.177  13.784 -13.286                       H
ATOM     67  CA  LEU A 474     -18.070   2.266 -12.702                       C
ATOM     68  H   LEU A 474     -17.333   2.432 -13.485                       H
ATOM     69  H   LEU A 474     -17.732   2.579 -11.715                       H
ATOM     70  HA  LEU A 474     -18.300   1.200 -12.671                       H
ATOM     71  C   LEU A 474     -19.375   3.014 -13.025                       C
ATOM     72  O   LEU A 474     -19.357   4.106 -13.616                       O
ATOM     73  N   HIS A 475     -20.504   2.464 -12.571                       N
ATOM     74  H   HIS A 475     -20.456   1.556 -12.103                       H
ATOM     75  CA  HIS A 475     -21.827   3.074 -12.708                       C
ATOM     76  HA  HIS A 475     -21.730   4.129 -12.467                       H
ATOM     77  C   HIS A 475     -22.814   2.469 -11.699                       C
ATOM     78  O   HIS A 475     -22.443   1.557 -10.958                       O
ATOM     79  CB  HIS A 475     -22.305   2.907 -14.169                       C
ATOM     80  HB2 HIS A 475     -21.554   3.310 -14.848                       H
ATOM     81  HB3 HIS A 475     -23.212   3.490 -14.324                       H
ATOM     82  CG  HIS A 475     -22.606   1.485 -14.561                       C
ATOM     83  ND1 HIS A 475     -23.870   0.970 -14.748                       N
ATOM     84  HD1 HIS A 475     -24.736   1.531 -14.679                       H
ATOM     85  CE1 HIS A 475     -23.768  -0.352 -14.961                       C
ATOM     86  HE1 HIS A 475     -24.596  -1.043 -15.071                       H
ATOM     87  NE2 HIS A 475     -22.462  -0.675 -14.949                       N
ATOM     88  HE2 HIS A 475     -22.071  -1.623 -14.992                       H
ATOM     89  CD2 HIS A 475     -21.715   0.460 -14.705                       C
ATOM     90  HD2 HIS A 475     -20.639   0.517 -14.563                       H
ATOM     91  N   SER A 476     -24.063   2.954 -11.674                       N
ATOM     92  H   SER A 476     -24.324   3.716 -12.289                       H
ATOM     93  CA  SER A 476     -25.123   2.403 -10.826                       C
ATOM     94  HA  SER A 476     -25.944   3.115 -10.828                       H
ATOM     95  CB  SER A 476     -25.671   1.110 -11.447                       C
ATOM     96  HB3 SER A 476     -24.857   0.506 -11.846                       H
ATOM     97  HB2 SER A 476     -26.187   0.532 -10.679                       H
ATOM     98  OG  SER A 476     -26.611   1.392 -12.466                       O
ATOM     99  H   SER A 476     -26.148   1.616 -13.276                       H
ATOM    100  C   SER A 476     -24.674   2.243  -9.360                       C
ATOM    101  O   SER A 476     -24.525   1.122  -8.863                       O
ATOM    102  N   TYR A 477     -24.443   3.376  -8.684                       N
```

```
ATOM    103  H   TYR A 477     -24.627   4.254  -9.166                   H
ATOM    104  CA  TYR A 477     -24.157   3.486  -7.250                   C
ATOM    105  HA  TYR A 477     -23.432   2.720  -6.976                   H
ATOM    106  H   TYR A 477     -23.712   4.464  -7.074                   H
ATOM    107  H   TYR A 477     -25.055   3.323  -6.655                   H
END
```

## 3. Explanation of the reported distance notation

The distance values used throughout the paper are measured from atom C45 of the 3CJ4 ligand (of the acid moiety) to the NE atom of Arg422, which is located at the bottom of the pocket. This is the case for both the 3CJ4 systems and the 3CJ2 systems, despite the C45 atom and the whole succinate moiety not existing for the 3CJ2 systems. Only the imaginary axis going from NE in Arg422 towards the position of C45 from the succinic acid allowed for the ligand to move away from the allosteric site without crashing with other residues or structures. This imaginary axis can be seen highlighted in yellow in Figure S5. For the 3CJ2 system progression, the coordinate is still C45-NE of Arg422 but then the succinate atoms were deleted and the nitrogen of aniline was just capped with two hydrogens. This way of working followed from the fact that only the axis Arg422NE-C45 of succinate had the exact direction for exiting the allosteric site without crashing with other residues. Since C45 is located near the top of the ligand and interacts with the outer parts of the allosteric site, the distance values towards the bottom of the allosteric site were too high and not representative of the distance from the bottom of the ligand (i.e. the bromine in the bromo-aryl moiety) to the bottom of the allosteric site (Arg422). Figure S5 shows an example of this. Although the Arg422NE-C45 distance is 11.3 Å, the distance from the actual bottom of the ligand (the bromine) is actually 5.5 Å. To couple these two values (11.3 Å and 5.5 Å), a value of 6 Å was subtracted from each C45-Arg422NE distance value in order to make the reported distances (5.3 Å in this case) a better representation of the real distance from the bottom of the drug to the pocket, as depicted in Figure S5 with green. Tables S2 and S3 report the distances for all the frames used in this work.

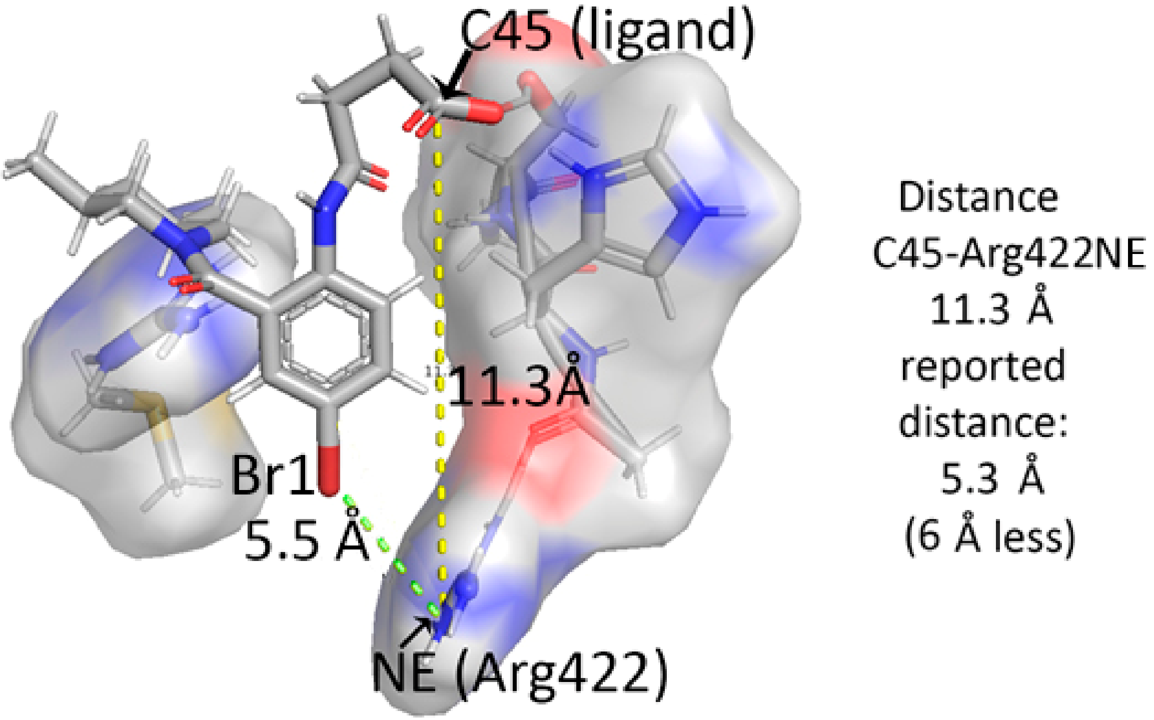


**Figure S5**. Diagram depicting the reported distances from the ligand to the bottom of the pocket. The atom C45 in the succinate lies directly above the bottom of the allosteric site represented by atom NE in Arg422. The distance between these two atoms is not an accurate representation of the separation of the bottom of the ligand (bromine atom) to the pocket, which is why 6 Å were subtracted from the distance value. The distance from Arg422 NE to Br1 in the ligand is included (5.5 Å), shown to be very similar in value to the reported 5.3 Å, and a better representation of the distance between the bottom of the allosteric site and the bottom of the ligand.

| **3CJ4 system** | | | | | | | | | | | | | | | | | |
|---|---|---|---|---|---|---|---|---|---|---|---|---|---|---|---|---|---|
| Configuration | 1 | 2 | 3 | 4 | 5 | 6 | 7 | 8 | 9 | 10 | 11 | 12 | 13 | 14 | 15 | 16 | 17 |
| Distance C45-NEArg422 [Å] | 9 | 9.5 | 9.7 | 9.9 | 10.3 | 10.5 | 10.9 | 11.1 | 11.3 | 11.5 | 11.9 | 15 | 16 | 17 | 18 | 20 | 28 |
| Reported distance in text [Å] | 3 | 3.5 | 3.7 | 3.9 | 4.3 | 4.5 | 4.9 | 5.1 | 5.3 | 5.5 | 5.9 | 9 | 10 | 11 | 12 | 14 | 22 |
| Actual distance from Br to NEArg422 [Å] | 2.66 | 2.82 | 2.91 | 3.01 | 3.23 | 3.36 | 3.63 | 3.77 | 3.92 | 4.07 | 4.39 | 7.14 | 8.08 | 9.03 | 9.99 | 11.94 | 19.82 |

**Table S2**. Distance notation used in this work for the 17 configurations of 3CJ4.

| **3CJ2 system** | | | | | | | | | | | | | | | | | | | | | | |
|---|---|---|---|---|---|---|---|---|---|---|---|---|---|---|---|---|---|---|---|---|---|---|
| Configuration | 1 | 2 | 3 | 4 | 5 | 6 | 7 | 8 | 9 | 10 | 11 | 12 | 13 | 14 | 15 | 16 | 17 | 18 | 19 | 20 | 21 | 22 |
| Distance C45-NEArg422 [Å] | 8.8 | 9 | 9.3 | 9.5 | 9.9 | 10 | 10.5 | 10.7 | 10.9 | 11.1 | 11.3 | 11.7 | 11.9 | 12.2 | 12.5 | 13 | 13.5 | 14 | 14.5 | 16 | 17 | 18 |
| Reported distance in text [Å] | 2.8 | 3 | 3.3 | 3.5 | 3.9 | 4 | 4.5 | 4.7 | 4.9 | 5.1 | 5.3 | 5.7 | 5.9 | 6.2 | 6.5 | 7 | 7.5 | 8 | 8.5 | 10 | 11 | 12 |
| Actual distance from Br to NEArg422 [Å] | 2.62 | 2.66 | 2.75 | 2.82 | 3.01 | 2.06 | 3.36 | 3.49 | 3.63 | 3.77 | 3.92 | 4.23 | 4.39 | 4.64 | 4.89 | 5.32 | 5.76 | 6.21 | 6.67 | 8.08 | 9.03 | 9.99 |

**Table S3**. Distance notation used in this work for the 22 configurations of 3CJ2.

## 4. REG ranking graphs

### 4.1 The 3CJ4 System

The first segment of 3CJ4 describes a sharp decrease in energy as the distance increases from very small values to that of the global minimum (see Figure 4a of the main text). Figure S6 shows the atoms that are closest to each other in the closest configuration. Some atoms shown are so close that the program Jmol depicts them as covalently bonded. These same atoms, particularly O47 (from the succinic acid chain of the ligand) and H91 (from His475), obtain a more negative IQA energy as the distance increases towards the global minimum, as can be deduced from their positive REG values shown in Table 2. Table 3 of the main text shows that, for O47, it is mainly the sterics ($E_{self}$) of the compressed atoms that drives the system while in segment 1. However, Table 3 also shows that the covalent component of the O47-H91 interaction (shown in Figure S6a) is against the separation towards the global minimum. These interactions are an example of how a single atom can have interactions both in favour of and against the system's energetic behaviour. The covalent interaction between H21 from the ligand and S69 from Met423 show a similar case. Despite H21 and S69 clearly following the system's energetic behaviour, becoming more negative as distance increases, the covalent component of the H21-S69 interaction clearly becomes less negative with the ligand-pocket separation.

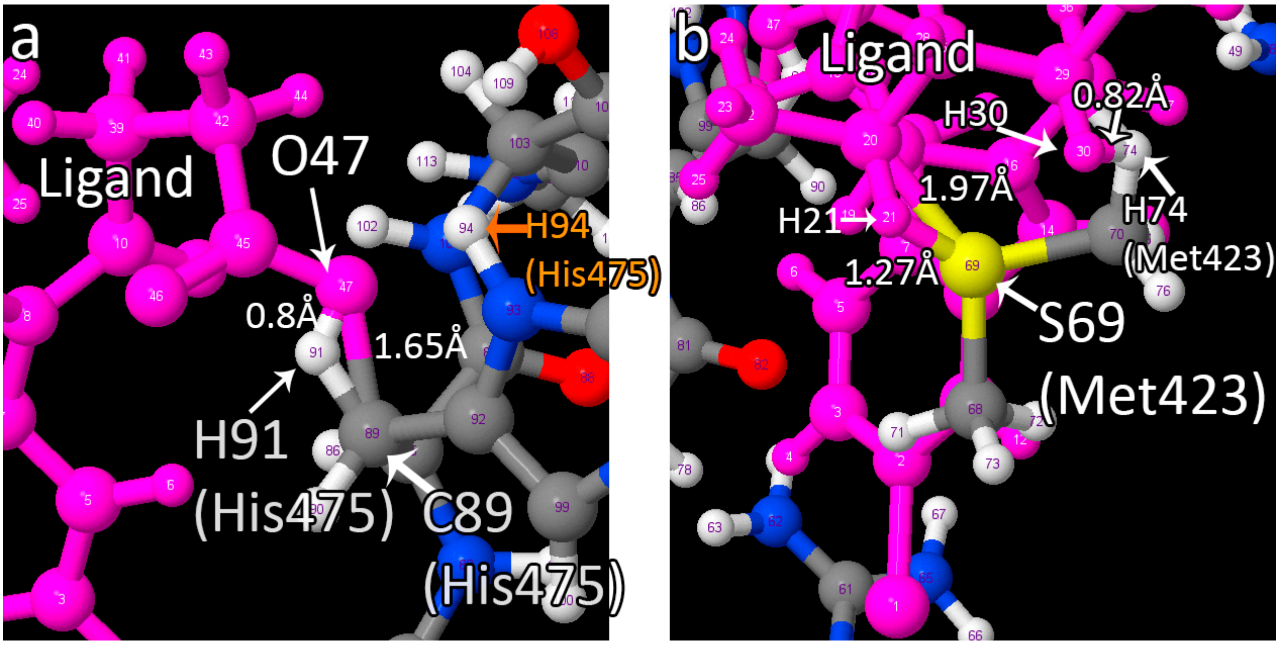


**Figure S6**. Diagram highlighting the atoms that clash the most between the ligand and the pocket in the closest configuration of 3CJ4. The ligand is shown in magenta and the pocket in CPK colours. The nuclei of several atoms are so close that JMOL depicts them as forming a covalent bond. Both panels show the system from a different point of view. In (a), atom O47 in the ligand appears very close to both H91 and C89 in His475, and their total IQA energy becomes more negative as the system approaches the global minimum. Atom H94 in the imidazole of His475 shows the opposite behaviour, that is, this atom adopts a less negative energy value as the system approaches the global minimum. In (b), H21 and H30 in the ligand appear very near S69 and H76 of Met423, respectively.

## 4.2 The 3CJ2 system

The following tables are a collection of several rankings that complete the picture in the main text for the segments of system 3CJ2. With the exception of Table S4, showing the *A-A′* ranking for the 3CJ2 segment 1, these tables clarify the type of IQA energies that contribute the most to the system's behaviour, but they should not be used as a first approach to do such analysis. The initial information that should be considered when analysing these tables explain can be extracted from the *A-A′* ranking of each segment

| Atom | Location | Partial charge | REG | Pearson Correlation Coefficient |
|---|---|---|---|---|
| S59 | Met423 | Neutral | 0.254 | 0.996 |
| H64 | Met423 | Neutral | 0.146 | 0.989 |
| H30 | Ligand piperidine, interacting directly with Met423 | Neutral | 0.132 | 0.997 |
| H21 | Ligand piperidine, interacting directly with Met423 | Neutral | 0.081 | 0.904 |
| N16 | Ligand piperidine, interacting directly with Met423 | Neutral | 0.043 | 0.986 |
| C60 | Met423 | Neutral | 0.038 | 0.920 |
| C20 | Ligand piperidine, interacting directly with Met423 | Neutral | 0.035 | 0.969 |
| H65 | Met423 | Neutral | 0.035 | 0.977 |
| C35 | Ligand piperidine, interacting directly with Met423 | Neutral | 0.034 | 0.940 |
| […] | […] | […] | […] | […] |
| N45 | Arg501 | Negative | -0.009 | -0.821 |
| N38 | Arg501 (hydrogen donor) | Negative | -0.012 | -0.837 |
| C14 | Ligand tertiary amide (neighbour of hydrogen acceptor) | Positive | -0.012 | -0.947 |

**Table S4**. The top atoms (*A-A′* ranking) by absolute REG values for segment 1 of 3CJ2. The ranking shows a strong contribution from the atoms of Met423 and the piperidine moiety from the 3CJ2 ligand.

Table S5 summarises the top pairwise interactions (*A*-*B* ranking) for segment 1 of the 3CJ2 system when the ligand and pocket are too close. It is very interesting to see the $E_{self}$ terms as the most important interactions that follow the system's behaviour, getting a lower energy as the distance between ligand and pocket increases, while the electrostatic and covalent character of some interactions between the ligand and the pocket work against the distance increase. Of course, the $E_{self}$ contributions are stronger. Note that the interactions involve mainly Met423, which is already crowded by the piperidine of the ligand.

| **Energy term** | **Location of A and of B, separated by a comma** | **REG** | **Pearson Correlation Coefficient** |
|---|---|---|---|
| Eself_s59 | Met423 | 0.381 | 0.998 |
| Eself_n16 | Ligand, interacting directly with Met423 | 0.169 | 0.999 |
| Eself_c60 | Met423 | 0.154 | 0.988 |
| Eself_h64 | Met423 | 0.132 | 0.979 |
| Eself_h21 | Ligand, interacting directly with Met423 | 0.132 | 0.989 |
| Vcl_pair_c14_s59 | Ligand (C14), Met423(S59) | 0.111 | 0.993 |
| Eself_h30 | Ligand, interacting directly with Met423 | 0.107 | 0.964 |
| Eself_c29 | Ligand, interacting directly with Met423 | 0.100 | 0.983 |
| Vxc_Pair_c60_h64 | Met423, Met423 | 0.081 | 0.988 |
| Vcl_Pair_o15_h39 | Ligand (O15), Met423 (H39) | 0.079 | 0.857 |
| Vcl_Pair_n16_c60 | Ligand (N16), Met423 (C60) | 0.076 | 0.998 |
| […] | […] | […] | […] |
| Vxc_Pair_c29_h64 | Ligand (C29), Met423 (H64) | -0.080 | -0.99 |
| Vcl_Pair_c14_c60 | Ligand (C14), Met423 (C60) | -0.083 | -0.998 |
| Vcl_Pair_o15_n38 | Ligand (O15), Ligand (N38) | -0.083 | -0.900 |
| Vxc_Pair_n16_s59 | Ligand (N16), Met423 (S59) | -0.086 | -0.997 |
| Vxc_Pair_c20_s59 | Ligand (C20), Met423 (S59) | -0.089 | -0.999 |
| Vcl_Pair_n16_s59 | Ligand (N16), Met423 (S59) | -0.126 | -0.989 |
| Vxc_Pair_h21_s59 | Ligand (H21), Met423 (S59) | -0.165 | -0.982 |

**Table S5**. The top pairwise interactions (*A*-*B* ranking) for segment 1 of 3CJ2, in favour of and against the system's energetic behaviour.

Table S6 summarises the *A-B* ranking for segment 2 of 3CJ2. Having just left the optimal distance for the hydrogen bond, the total energy becomes more positive. The top contributors in favour of the system's behaviour are precisely the oppositely charged atoms of the hydrogen bond. Evidence supports the concept of the "neighbour effect" because the interactions between the covalently bonded N38 and H39 in Arg501 are shown as opposite contributors, becoming more negative once there is no hydrogen bond. Regarding the O15 from the ligand, it can be seen that, while its interactions with H39 and C41 from the Arg501 indeed follow the system's behaviour and become less negative as distance increases, the same O15 has interactions with other atoms from Arg501 but this time the energy becomes more negative as distance increases, showing that the same atom can have interactions in opposite senses with atoms of the same residue, and this is expected in the partial charges that allow the forming of hydrogen bonds.

| **Energy term (A_B)** | **Location of A and of B, separated by a comma** | **Partial charge** | **REG** | **Pearson Correlation Coefficient** |
|---|---|---|---|---|
| Vcl_Pair_o15_h39 | Ligand (O15), Arg501 (H39) | Negative, positive | 9.993 | 0.982 |
| Vcl_Pair_n38_h39 | Arg501, Arg501 | Negative, positive | 8.198 | 0.973 |
| Vcl_Pair_o15_c41 | Ligand (O15), Arg501 (C41) | Negative, positive | 8.186 | 0.985 |
| Vcl_Pair_c14_n38 | Ligand (C14), Arg501 (N38) | Positive, negative | 5.988 | 0.974 |
| Vcl_Pair_c14_n42 | Ligand (C14), Arg501 (N42) | Positive, Negative | 4.095 | 0.990 |
| Vcl_Pair_n8_c77 | Ligand (N8), His475 backbone (C77) | Negative, positive | 3.770 | 0.991 |
| Vcl_Pair_n8_c41 | Ligand (N8), Arg501 (C41) | Negative, positive | 3.733 | 0.992 |
| Vcl_Pair_n8_c51 | Ligand (N8), Arg422 (C51) | Negative, positive | 3.554 | 0.987 |
| Vcl_Pair_n8_c71 | Ligand (N8), His475, backbone (C71) | Negative, Positive | 3.467 | 0.990 |
| Vcl_Pair_o15_c51 | Ligand (O15), Arg422(C51) | Negative, Positive | 3.392 | 0.990 |
| […] | […] | […] | […] | […] |
| Eself_h39 | Arg501 | | -4.078 | -0.974 |
| Vcl_Pair_n38_h39 | Arg501, Arg501 | Negative, positive | -4.461 | -0.991 |
| Vxc_Pair_n38_h39 | Arg501, Arg501 | Negative, positive | -4.524 | -0.972 |
| Vcl_Pair_c14_c41 | Ligand (C14), Arg501 (C41) | Positive, positive | -5.012 | -0.979 |
| Vcl_Pair_o15_n42 | Ligand (O15), Arg501 (N42) | Negative, negative | -6.299 | -0.992 |
| Vcl_Pair_c14_h39 | Ligand (C14), Arg501 (H39) | Positive, Positive | -6.516 | -0.982 |
| Vcl_Pair_o15_n38 | Ligand (O15), Arg501 (N38) | Negative, negative | -9.321 | -0.976 |

**Table S6**. The top pairwise interactions (*A-B* ranking) for segment 2 of 3CJ2, in favour of and against the system energetic behaviour.

Meanwhile, the *A-B* ranking for segment 4, shown in Table S7, confirms that at this point the top representative terms are all electrostatic interactions ($V_{cl}$), and that the atoms involved in the hydrogen bond O15, C14, N38 show a preponderance. Segments 2 and 4 from 3CJ2 are equivalent to segment 2 in 3CJ4.

| Energy term (A_B) | Location of A and of B, separated by a comma | Partial charge | REG | Pearson Correlation Coefficient |
|---|---|---|---|---|
| Vcl_Pair_o15_c41 | Ligand, Arg501 | Negative, positive | 11.082 | 1 |
| Vcl_Pair_c14_n38 | Ligand, Arg501 | Positive, negative | 9.537 | 1 |
| Vcl_Pair_c14_n42 | Ligand, Arg501 | Positive, negative | 5.971 | 1 |
| Vcl_Pair_n8_c41 | Ligand, Arg501 | Negative, positive | 5.658 | 1 |
| Vcl_Pair_n16_c41 | Ligand, Arg501 | Negative, positive | 5.629 | 1 |
| Vcl_Pair_c14_n45 | Ligand, Arg501 | Positive, negative | 5.410 | 1 |
| Vcl_Pair_n8_c77 | Ligand, His475 | Negative, positive | 5.098 | 1 |
| […] | […] | […] | […] | […] |
| Vcl_Pair_c14_h40 | Ligand, Arg501 | Positive, positive | -4.859 | -1 |
| Vcl_Pair_c14_h39 | Ligand, Arg501 | Positive, positive | -4.968 | -1 |
| Vcl_Pair_n8_n38 | Ligand, Arg501 | Negative, negative | -5.001 | -1 |
| Vcl_Pair_n8_n91 | Ligand, Arg501 | Negative, negative | -5.656 | -1 |
| Vcl_Pair_o15_n45 | Ligand, Arg501 | Negative, negative | -5.871 | -1 |
| Vcl_Pair_o15_n42 | Ligand, Arg501 | Negative, negative | -6.305 | -1 |
| Vcl_Pair_n8_o98 | Ligand, Ser476 | Negative, negative | -6.390 | -1 |
| Vcl_Pair_o15_n38 | Ligand, Arg501 | Negative, negative | -10.232 | -1 |
| Vcl_Pair_c14_c41 | Ligand, Arg501 | Positive, positive | -10.318 | -1 |

**Table S7**. The top pairwise interactions (*A-B* ranking) for segment 4 of 3CJ2, in favour of and against the system energetic behaviour. The most important interactions appear between the atoms that form the hydrogen bond, as well as electrostatic attraction between different partial charges, and repulsion between same-sign partial charges.

For segment 3, the *A-B* pairwise ranking does not explain the importance of the bromine, but the ranking is shown here in Table S8 for the sake of completeness. All the top terms have to do with the hydrogen bonds and the neighbor effect. A preponderance of electrostatic $V_{cl}$ interactions between ligand atoms such as O15, C14, N8 and atoms in Arg501 are the most important pairwise interactions. In fact, this Table S8 is quite similar to Table S7, albeit in an opposite sense (because of the opposite system wave function energy system's behaviour in segments 3 and 4 of 3CJ2). *This shows that the electrostatic interactions are so strong that they can mask other effects*. However, because Table 7 in the main text shows the importance of bromine, we generated an *A-B* ranking for bromine interactions only. This special *A-B* ranking of bromine pairwise interactions is reported in Table 8 in the main text.

| **Energy term (A_B)** | **Location of A and of B, separated by a comma** | **Partial charge** | **REG** | **Pearson Correlation Coefficient** |
|---|---|---|---|---|
| Vcl_Pair_o15_n38 | Ligand, Arg501 | Negative, negative | 15.479 | 0.970 |
| Vcl_Pair_c14_c41 | Ligand, Arg501 | Positive, positive | 11.974 | 0.956 |
| Vcl_Pair_o15_n42 | Ligand, Arg501 | Negative, negative | 8.634 | 0.966 |
| Vcl_Pair_n8_n91 | Ligand, Ser476 | Negative, negative | 7.077 | 0.963 |
| Vcl_Pair_o15_n45 | Ligand, Arg501 | Negative, negative | 6.909 | 0.958 |
| Vcl_Pair_c14_h39 | Ligand, Arg501 | Positive, positive | 6.461 | 0.972 |
| Vcl_Pair_n8_n38 | Ligand, His475 | Negative, negative | 6.030 | 0.960 |
| Vcl_Pair_n16_n38 | Ligand, Arg501 | Negative, negative | 5.411 | 0.953 |
| Vcl_Pair_n8_n83 | Ligand, His475 | Negative, negative | 4.843 | 0.947 |
| Vcl_Pair_c14_h40 | Ligand, Arg501 | Positive, positive | 4.776 | 0.957 |
| […] | […] | […] | […] | […] |
| Vcl_Pair_c14_n45 | Ligand, Arg501 | Positive, negative | -5.510 | -0.949 |
| Vcl_Pair_n16_c41 | Ligand, Arg501 | Negative, positive | -5.920 | -0.951 |
| Vcl_Pair_n8_c41 | Ligand, Arg501 | Negative, positive | -6.466 | -0.957 |
| Vcl_Pair_c14_n42 | Ligand, Arg501 | Positive, negative | -7.102 | -0.959 |
| Vcl_Pair_o15_h39 | Ligand, Arg501 | Negative, positive | -8.039 | -0.979 |
| Vcl_Pair_c14_n38 | Ligand, Arg501 | Positive, negative | -12.081 | -0.961 |
| Vcl_Pair_o15_c41 | Ligand, Arg501 | Negative, positive | -15.021 | -0.965 |

**Table S8**. The top pairwise interactions (*A-B* ranking) for segment 3 of 3CJ2, in favour of and against the system energetic behaviour. No top term involves the bromine atom.

## 5. Interacting Quantum Fragments Ligand-Pocket Analysis

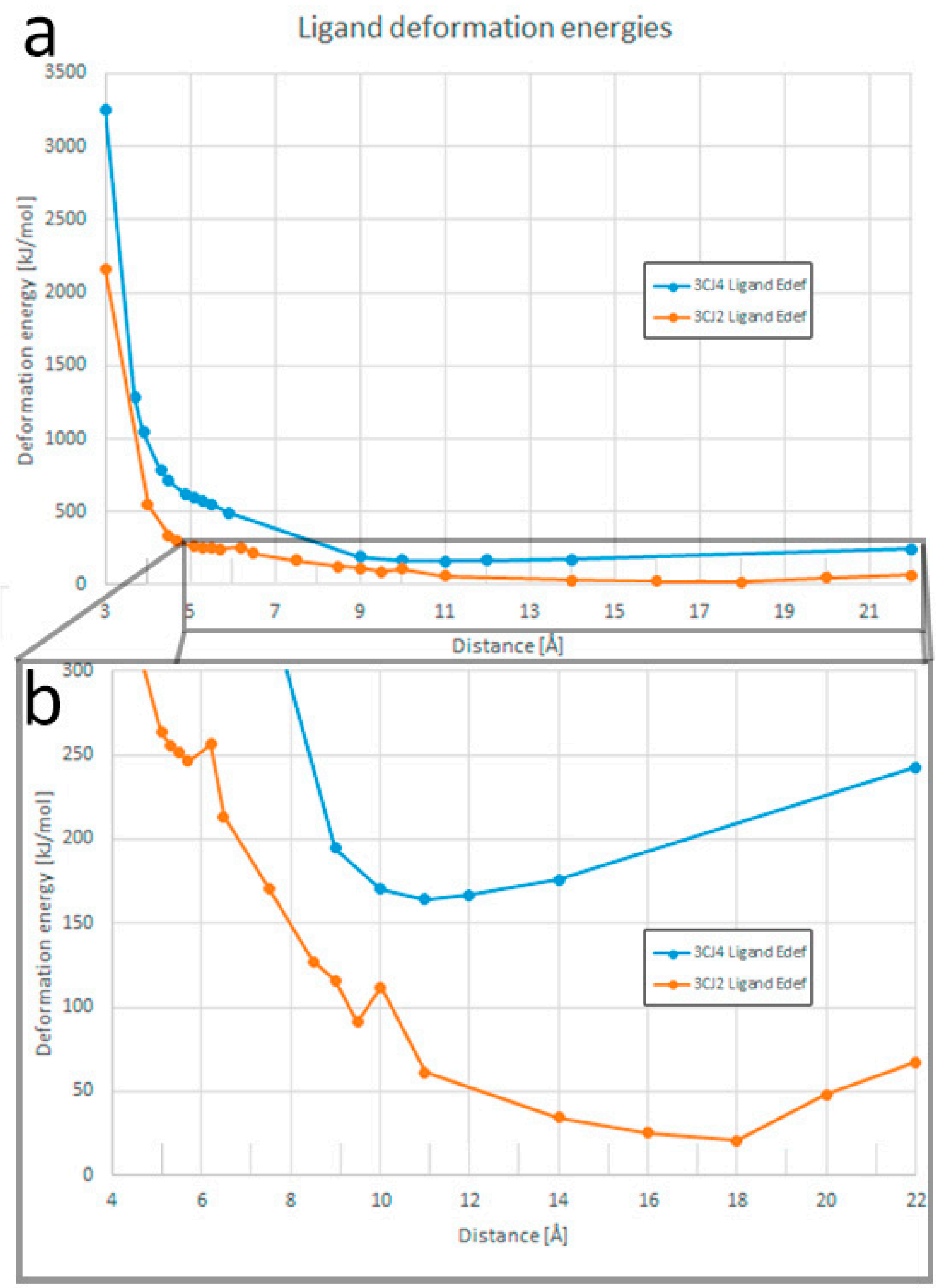


**Figure S7**. The deformation energies for the 3CJ4 and 3CJ2 ligands across the studied configurations. The deformation energies were calculated using the formula $E^{A}_{def} = E^{A}_{self\,(\text{in system})} - E^{A}_{self\,(\text{free})}$. Panel (a) shows that, at an initial ligand-pocket distance of 3 Å, the deformation energy is high as a consequence of the short ligand-pocket distance but quickly diminishes as the distance increases. Panel (b) is an amplified version of (a) at longer distances showing that, as the ligand-pocket distance increases, the deformation energies decrease. However, despite a separation greater than 20 Å, they do not reach zero. Instead, an increase in deformation energy is observed after a minimum is reached at 11 Å for 3CJ4 and 18 Å for 3CJ2, respectively. The deformation energy of the ligand becomes zero when the steric energy in the ligand-pocket system is the same as when the ligand is in the system by itself. It is intuitive to think that this would happen once the ligand-pocket distance is far enough. The obtained results suggest that a 22 Å ligand-pocket separation distance is still not enough for the ligands acquire a deformation energy of zero.

In 3CJ2, the closest analysed frame, at a separation of 3 Å, shows an interaction energy of 576% the value at the global minimum at 5.3 Å. From this ligand-pocket interaction at 3 Å, 95% (547% of the global minimum) was $V_{xc}$. The total energy quickly decreases to 180% at 4 Å and 130% at 4.5 Å. The closest frame in 3CJ4 was calculated also at a distance of 3 Å but shows an 80% covalent character. Since the 3CJ4 ligand benefits from more polar interactions with the NS5B protein, the total interaction is more negative. At short range, 3 Å from the bottom of the site, the interaction corresponds to 232% that of the interaction energy in the global minimum. One must keep in mind that the ligand-pocket interaction in 3CJ4 is always stronger than the equivalent interaction in 3CJ2, by a factor that starts at 1.6 at the closest studied configurations (3 Å). This factor increases with distance until it steadies, reaching a plateau at separations between 4.7 to 11 Å, where the 3CJ4 interaction is 3.5-4 times stronger. In distances longer than 11 Å, where the interaction of the pocket with the 3CJ2 ligand is minimal, the 3CJ4 interaction is up to 17 times stronger than the corresponding interaction from 3CJ2. Figure S8 shows the corresponding graph. As the distance between the ligand and pocket increases, the interaction energy decreases to 1.5% (3CJ2) or 6% (3CJ4) of the interaction at the global minimum at a separation of 22 Å.

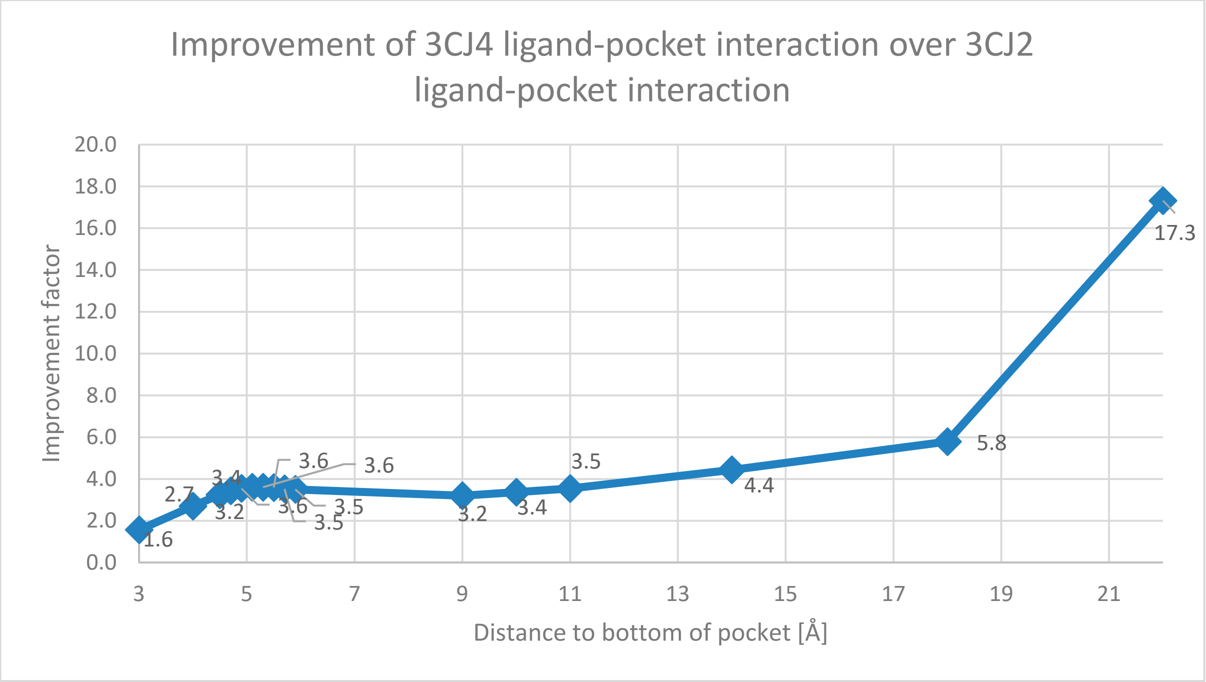


**Figure S8**. Improvement of $E_{inter}$ between the whole 3CJ4 ligand and the whole pocket compared with the full $E_{inter}$ between the whole 3CJ2 ligand and the whole pocket. Starting at a factor of 1.6, the value plateaus at 3.5 to 4 times the $E_{inter}$ value for 3CJ2, for the distances ranging from the respective global minimum to slightly further than 11 Å. At this point, the interaction of 3CJ2 with the pocket is quite small compared to the corresponding interaction of 3CJ4.
